# Gompertz Law‐Based Biological Age (GOLD BioAge): A Simple and Practical Measurement of Biological Ageing to Capture Morbidity and Mortality Risks

**DOI:** 10.1002/advs.202501765

**Published:** 2025-07-02

**Authors:** Meng Hao, Hui Zhang, Jingyi Wu, Yaqi Huang, Xiangnan Li, Meijia Wang, Shuming Wang, Jiaofeng Wang, Jie Chen, Zhi jun Bao, Li Jin, Xiaofeng Wang, Zixin Hu, Shuai Jiang, Yi Li

**Affiliations:** ^1^ Department of Geriatric Medicine, Huadong Hospital, Shanghai Medical College Fudan University Shanghai 200040 China; ^2^ State Key Laboratory of Genetic Engineering, Collaborative Innovation Center for Genetics and Development, School of Life Sciences and Human Phenome Institute Fudan University Shanghai 200438 China; ^3^ Fudan Zhangjiang Institute Shanghai 200120 China; ^4^ School of Global Health, Chinese Center for Tropical Diseases Research Shanghai Jiao Tong University School of Medicine Shanghai 200025 China; ^5^ Artificial Intelligence Innovation and Incubation Institute, Fudan University, Shanghai Academy of Artificial Intelligence for Science, Shanghai, China Fudan University Shanghai 200438 China; ^6^ Department of Gerontology, Huadong Hospital, Shanghai Medical College Fudan University Shanghai 200040 China; ^7^ Department of Vascular Surgery, Shanghai Key Laboratory of Vascular Lesion Regulation and Remodeling, Shanghai Pudong Hospital Fudan University Pudong Medical Center Shanghai 201399 China

**Keywords:** aging clocks, biological age, frailty index, metabolomics, proteomics

## Abstract

Biological age reflects actual ageing and overall health, but current ageing clocks are often complex and difficult to interpret, which limits their clinical application. This study introduces a Gompertz law‐based biological age (GOLD BioAge) model designed to simplify the assessment of ageing. We calculated GOLD BioAge using clinical biomarkers and found significant associations between the difference from chronological age (BioAgeDiff) and the risks of morbidity and mortality in the NHANES and UK Biobank. Using proteomics and metabolomics data, we developed GOLD ProtAge and MetAge, which outperformed the clinical biomarker models in predicting mortality and chronic disease risk in UK Biobank. Benchmark analyses demonstrated that the models outperformed common ageing clocks in predicting mortality across diverse age groups in both the NHANES and UK Biobank cohorts. Additionally, a simplified version called Light BioAge is created, which uses three biomarkers to assess ageing. The Light model reliably captured the mortality risk across three validation cohorts (CHARLS, RuLAS, and CLHLS). It significantly predicted the onset of frailty, stratified frail individuals, and collectively identified individuals at high risk of mortality. In summary, the GOLD BioAge algorithm provides a valuable framework for the assessment of ageing in public health and clinical practice.

## Graphical abstract

The Gompertz law‐based biological age metric (GOLD BioAge) introduces interpretable convenient and interpretable calculations to capture mortality, disease, and frailty risks. Its applications to proteomics and metabolomics (ProtAge and MetAge) demonstrate clinical potential to enhance aging evaluation and prevent age‐related diseases. A simplified version, Light BioAge, is developed and validated across independent datasets.

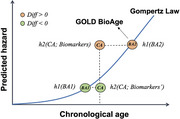

## 1 Introduction

Human ageing manifests as progressive physiological changes and a decline in physical and cognitive function that leads to an increased risk of mortality.^[^ ^1^ ^]^ There is significant heterogeneity among individuals during the ageing process,^[^ ^2^ ^]^ and chronological age may not accurately reflect the actual pace of ageing. Furthermore, because ageing is the primary risk factor for most chronic diseases, targeting the ageing process itself may delay multiple age‐associated diseases.^[^ ^3^ ^]^ Consequently, ageing assessments and treatments have the potential to predict and prevent functional decline and age‐related chronic diseases.^[^ ^4^ ^]^ Some routine clinical biomarkers serve as biomarkers for ageing and predict the risks of functional decline and mortality after adjusting for chronological age.^[^ ^5^ ^]^ Integrating these biomarkers into composite panels may offer a more comprehensive and powerful assessment of ageing than single biomarkers alone.

Biological age measures an organism's biological functioning compared with the expected level for a specific chronological age to reflect overall health.^[^ ^6^, ^7^ ^]^ Levine's phenotypic age, which integrates nine biomarkers with chronological age, can predict mortality more accurately than chronological age alone.^[^ ^8^ ^]^ Building on the concept of phenotypic age, Sheng et al. proposed PCAge to estimate biological age through linear dimensionality reduction; however, this method may be sensitive to outliers and thresholding effects.^[^ ^9^ ^]^ Wei et al. presented ENABLAge, which integrates machine‐learning models with explainable artificial intelligence to ensure high prediction accuracy.^[^ ^10^ ^]^ In addition to clinical ageing clocks, omics‐based ageing clocks hold significant promise as they capture more precise dynamic molecular interactions and pathways that are closely tied to the biological ageing process.^[^ ^11^ ^]^ Specifically, epigenetic biomarkers have been utilized extensively in DNA methylation ageing clocks, such as the Horvath Clock^[^ ^12^ ^]^ and GrimAge Clock.^[^ ^13^ ^]^ Furthermore, multi‐tissue aging clocks provide insights into the molecular changes that complex organisms undergo with age to offer detailed information about ageing and disease.^[^ ^11^, ^14^, ^15^ ^]^ Recently, proteomics and metabolomics data from large cohorts have been used to accelerate the development of plasma proteomic and metabolomic ageing clocks.^[^ ^16^, ^17^, ^18^, ^19^, ^20^, ^21^ ^]^ Proteomic aging clocks show promising accuracy in predicting mortality and multimorbidity,^[^ ^14^, ^16^ ^]^ Although these biological ageing clocks demonstrate excellent performance in predicting disease and mortality, however, their clinical translation remains limited because of the gap between scientific research and their application in clinical settings.^[^ ^22^ ^]^

The complexity of current models of ageing clocks combined with challenges related to interpretability, required features, and generalizability may hinder their translation into clinical settings. For example, despite the widespread adoption of DNA methylation clocks,^[^ ^12^ ^]^ the collection of biological samples and reliance on high‐throughput sequencing remain time intensive and costly. As public demand for personalized health tracking grows, it becomes critical to ensure the clinical relevance and practicality of ageing clocks. Improvements to these tools must generate actionable clinical insights while maintaining cost‐effectiveness, accessibility, and robustness across diverse populations. Although omics‐driven methods remain costly and technically demanding, aging clocks based on routine clinical biomarkers offer a practical solution for health care integration owing to their accessibility and widespread applicability. Addressing these clinical challenges will require refining computational methods and identifying robust biomarkers that balance precision with practicality.

The Gompertz law is one of the most widely used mathematical models for describing mortality. It effectively captures the exponential increase in the mortality hazard across adult ages, in line with empirical mortality data.^[^ ^23^ ^]^ The model's simplicity and flexibility allow for its wide application. For example, Levine's phenotypic age used the Gompertz model to estimate 10‐year mortality risk.^[^ ^8^ ^]^ Additionally, Kuo et al. employed the model's cumulative mortality risk to develop a proteomic ageing clock.^[^ ^19^ ^]^ The Gompertz model provides a theoretical basis for biological ageing clocks in clinical practice.

In this study, we develop a Gompertz law‐based biological age (GOLD BioAge) algorithm that utilizes the hazard function of the Gompertz distribution. This approach offers an easily calculated linear model that combines chronological age and routine biomarkers to link the deviation from chronological age to morbidity and mortality risks. We apply the GOLD BioAge algorithm to metabolomics and proteomics data from the UKB to investigate the algorithm's validity with omics‐based data. Moreover, we compare its predictive performance for mortality with common ageing clocks using data from the National Health and Nutrition Examination Survey (NHANES) and the UK Biobank (UKB). Finally, we refine and simplify GOLD BioAge as a light model and validate it across three independent Chinese cohorts: the China Health and Retirement Longitudinal Study (CHARLS), the Chinese Longitudinal Healthy Longevity Survey (CLHLS), and the Rugao Longevity and Ageing Study (RuLAS).

## 2 Results

### 2.1 Definition and Development of the GOLD BioAge Model

**Figure 1:**
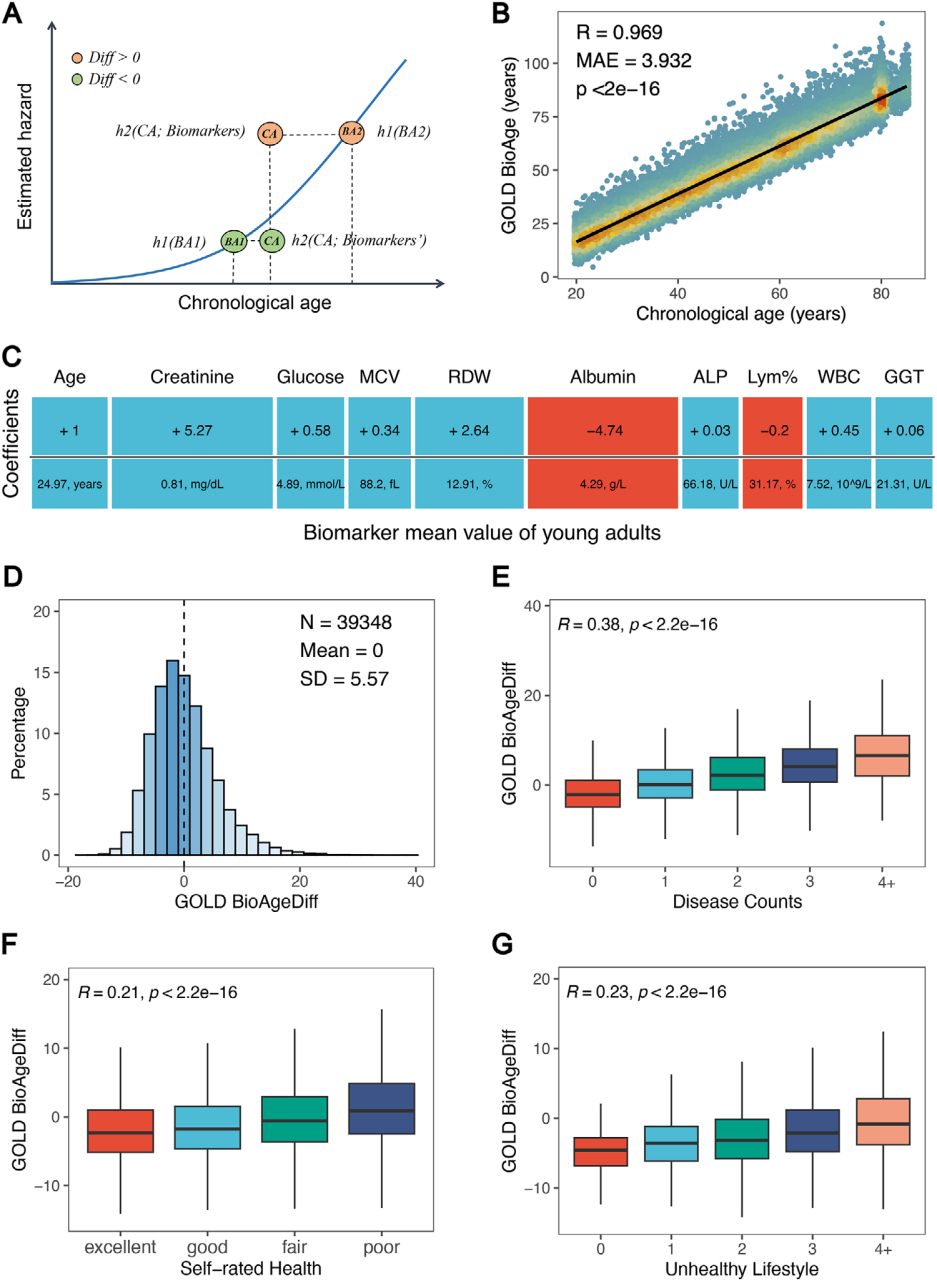
GOLD BioAge and its Association with Health‐Related Factors. Panel A illustrates the exponential relationship between mortality hazard and biological age (BA) and chronological age (CA). The “Diff” referred to the difference between GOLD BioAge and CA, termed GOLD BioAgeDiff. The scatter plot B) shows the strong correlation between GOLD BioAge (estimated biological age) and CA. The estimated coefficients for CA and biomarkers C), used to calculate GOLD BioAge, were displayed, with the mean biomarker values of young adults serving as the reference. Abbreviation: MCV: mean cell volume; RDW: red cell distribution width, ALP: alkaline phosphatase, LYM%: lymphocyte percent, WBC: white blood cell count, GGT: gamma glutamyl transferase (GGT). The distribution of GOLD BioAgeDiff in NHANES D). The correlations of GOLD BioAgeDiff with counts of age‐related chronic diseases E), self‐rated health F), and unhealthy lifestyles (G).

Biological age refers to the age that accurately reflects an individual's risk of mortality. Higher mortality risk corresponds to older biological age. Based on the Gompertz model, we linked chronological age and biomarkers to mortality hazard with an exponential distribution (Supplementary Methods, **Figure** **1**A). Consequently, the Gompertz law‐based biological age (GOLD BioAge) was estimated as the age that aligned with the joint mortality hazard derived from both chronological age and biomarkers. Thus, the GOLD Biological Age (GOLD BioAge) was derived as follows:

(1) $$GOLD\;BioAge=CA+\sum \beta_{i}*\mathrm{Biomaeke}r_{i}+\beta_{0}$$

with *Biomarker_i_* indicating the i‐th biomarker, β_*i*_ as its corresponding coefficient, and β_0_ as the constant.

The NHANES included 39,348 samples (49.5 ± 18.0 years old) with 26 biomarkers and chronological ages in the analysis. After feature selection was implemented via LASSO‐Cox regression (Figure S1, Supporting Information), we developed a clinical ageing clock, GOLD BioAge, based on chronological age and 9 biomarkers, which showed a strong correlation with chronological age (R = 0.969, Figure 1B). GOLD BioAge is the linear combination of chronological age, red blood cell distribution width (RDW), albumin (ALB), creatinine, etc (Figure 1C). It demonstrates how changes in specific biomarker values, such as RDW and ALB, contribute to biological age to provide an intuitive interpretation of biomarkers and ageing at the individual level.

### 2.2 GOLD BioAgeDiff as a Novel Ageing Metric

We introduced GOLD biological age difference (BioAgeDiff) as the difference between BioAge and chronological age to estimate the magnitude of how individuals’ biological age deviates from their chronological age (Figure 1A; Figure S2, Supporting Information). If the BioAgeDiff is greater/lower than 0, the person is older/younger than the CA. The BioAgeDiff, as a linear combination of biomarkers, establishes a clear relationship between changes in biomarkers and shifts in biological age. This calculation of BioAgeDiff facilitates understanding of how deviations in biomarkers from reference values affect biological age. The BioAgeDiff can be interpreted through the equation below:

(2) $$\begin{array}{ccc} & & GOLD\;BioAgeDiff\;\left(\Delta Age\right)\\ & & \;=\sum \beta_{i}*\left(\mathrm{Biomarke}r_{i}-\mathrm{Biomarke}r_{\mathrm{refi}\;}\right)+{\beta_{0}}^{\prime } \end{array}$$

where *Biomarker*_*refi*_is the reference value of the i‐th biomarker. For example, if an individual's blood glucose level increases by 1 mmol/L, BioAge increases by 0.58 years (Figure 1C).

Figure 1D shows the distribution of BioAgeDiff, which is close to a normal distribution (mean: 0, standard deviation (SD): 5.57). When major chronic diseases were considered, participants with comorbidities had greater BioAgeDiff values than those without chronic diseases (Figure 1E). Notably, individuals with four diseases were approximately 5 years older in BioAge. With regard to health status, a higher BioAgeDiff was found to be cross‐sectionally associated with poorer self‐rated health (Figure 1F). Additionally, unhealthy lifestyles, such as smoking and alcohol use, were associated with a greater BioAgeDiff (Figure 1G). The results of BioAgeDiff were validated in the UKB (Figure S3, Supporting Information).

**Figure 2:**
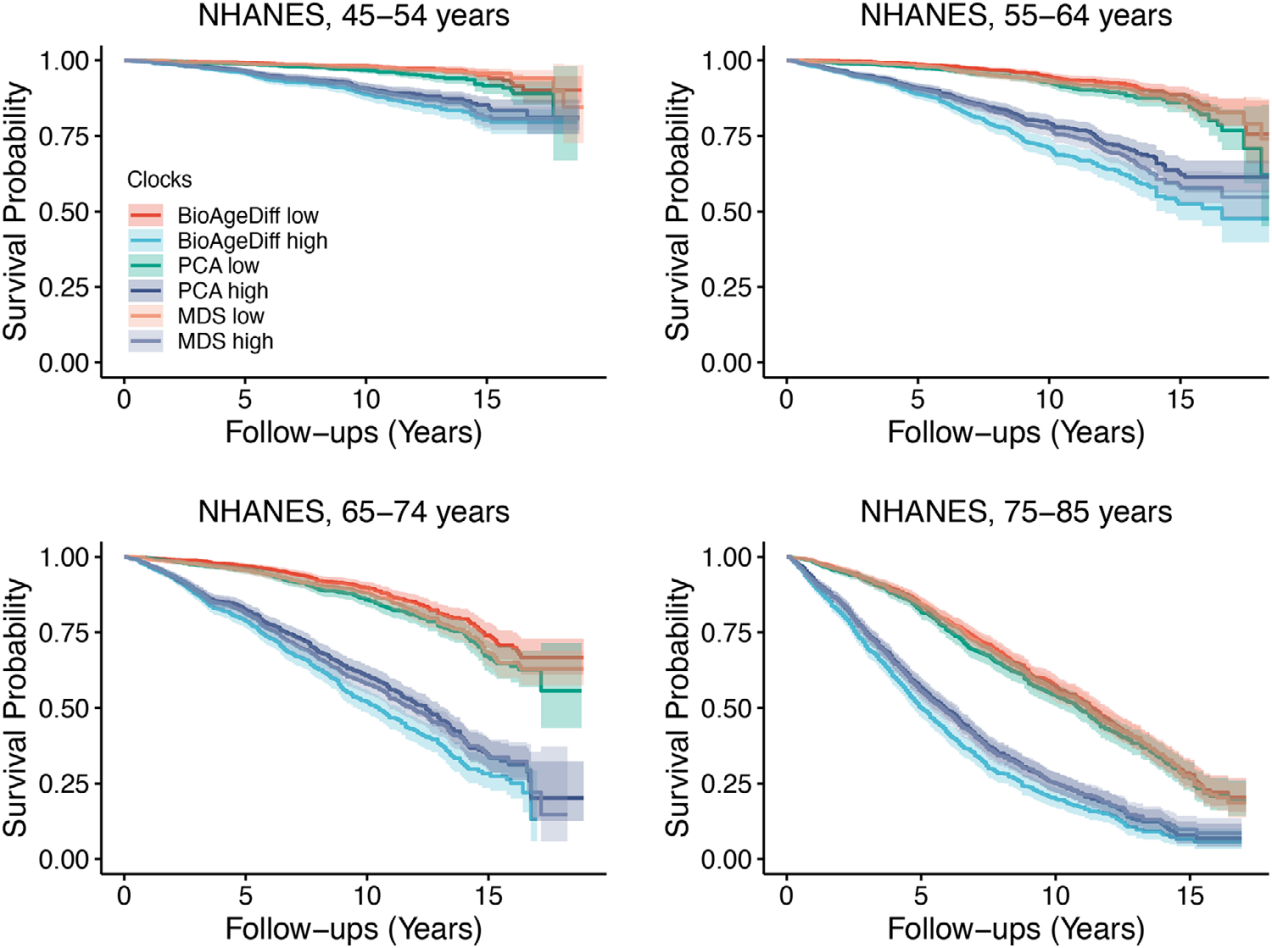
The associations of BioAgeDiff and risks of mortality. Survival plots for individuals categorized by BioAgeDiff, PCA age, and MDS in the NHANES cohort are presented. The high and low risk groups represent the top and bottom 25% of the age‐stratified population (ages 45–54, 55–64, 65–74, and 75–85 years). PCA: principal component analysis; MDS: Mahalanobis distance statistics.

BioAgeDiff was associated with risks of mortality in the NHANES and UKB (Table 1), with hazard ratios (HRs) of 1.155 (1.150–1.159) and 1.133 (1.131–1.135) per 1‐year increase, respectively. Survival curve analysis (**Figure** **2**) of 20‐year follow‐up data revealed that participants in the highest 25% of the BioAgeDiff groups had a steeper decline in survival probability than those in the lowest 25% of the groups, especially among middle‐aged and older age groups. For example, among individuals aged 65–74 years, approximately 74.9% of those in the high‐risk group died after approximately 16 years, whereas only approximately 30.2% of those in the low‐risk group died. BioAgeDiff can be considered a measure through linear dimension reduction or projection. Thus, we also compared the performance of BioAgeDiff with common metrics, including the Mahalanobis distance statistic^[^ ^24^, ^25^ ^]^(MDS) and principal component analysis^[^ ^26^ ^]^ (PCA). Among middle‐aged (45–64 years) and older (65–85 years) age groups, BioAgeDiff outperformed other linear metrics in identifying individuals at high risk of mortality (Figure 2).

### 2.3 Application of GOLD BioAge to Metabolomics and Proteomics

**Figure 3:**
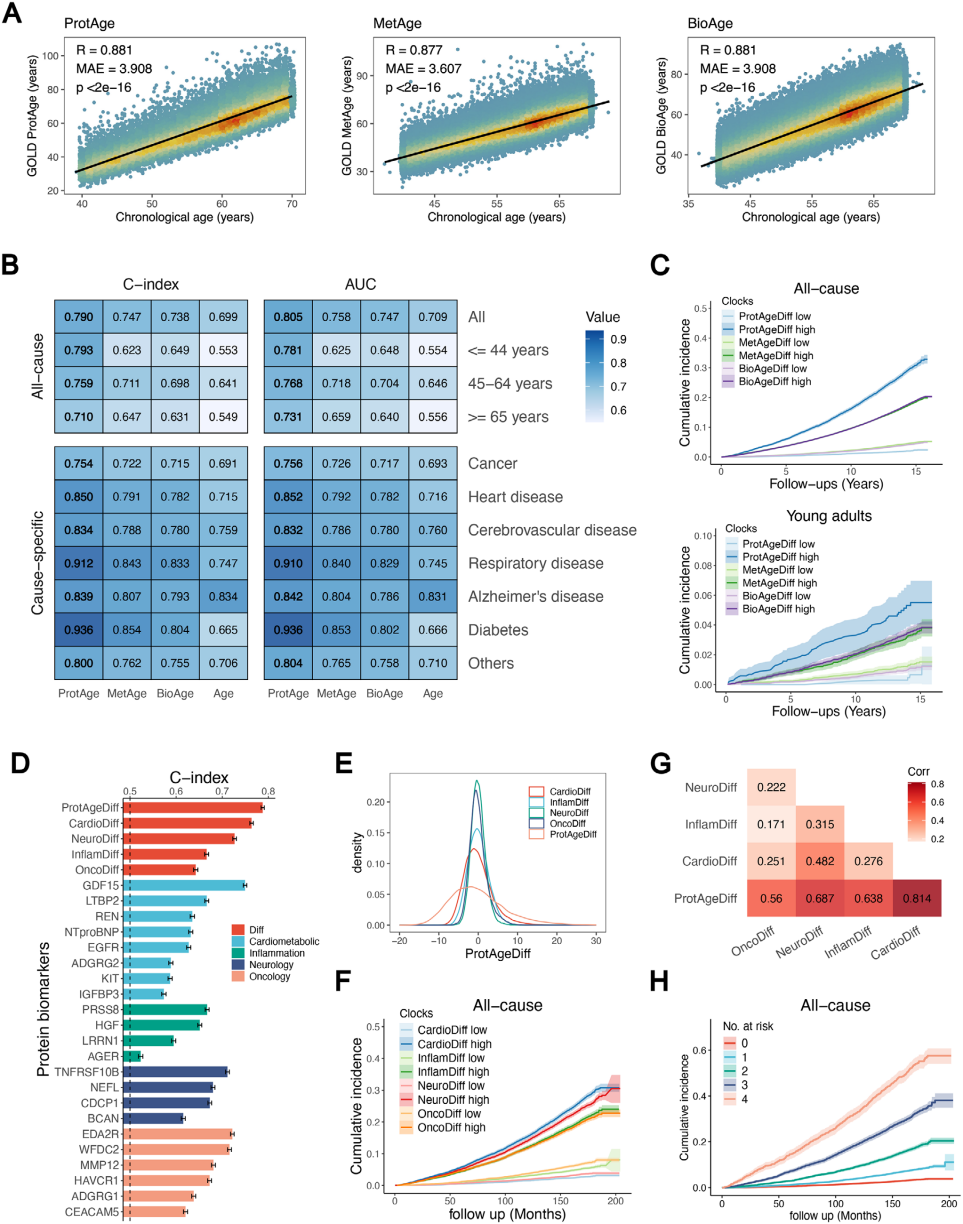
The associations of GOLD ProtAge, MetAge, and BioAge with mortality in UK Biobank. A) Correlations between the three aging clocks and chronological age. B) C‐index values from survival analysis and the AUC for 10‐year mortality prediction, comparing the three aging clocks and chronological age, with results for all‐cause (age‐stratified) and cause‐specific mortality. C) Survival curves for individuals classified by ProtAgeDiff, MetAgeDiff, and BioAgeDiff in the general population (top panel) and young adults (bottom panel, <45 years old). High and low risk groups are defined as the top and bottom 25% of the population. ProtAgeDiff, MetAgeDiff, and BioAgeDiff represent the differences between ProtAge, MetAge, and BioAge and chronological age, respectively. The C‐index for ProtAgeDiff and its subpanels and proteins are shown. ProtAgeDiff consisted of CardioDiff, InfamDiff, NeuroDiff, and OncoDiff, which were linear combinations of cardiometabolic, inflammatory, neurological, and oncological proteins. E) Density plots and G) a correlation heatmap (filled with Pearson correlation coefficients) of these subpanels are presented. F) Survival plots based on ProtAgeDiff subpanels and H) the risk score, which was the count of high‐risk factors derived from ProtAgeDiff subpanels.

We applied our algorithm to create the MetAge and ProtAge models on the basis of blood NMR metabolomics and proteomics data in the UKB, respectively. Like the clinical‐based BioAge, the omics‐based ageing clocks showed strong correlations with chronological age and age‐related factors (**Figure** **3**A; Figure S4, Supporting Information). ProtAge exhibited a significant ability to capture mortality risk that surpassed MetAge, clinical BioAge and chronological age (Figure 3B). For all‐cause mortality, ProtAge achieved a C‐index of 0.790, whereas MetAge and BioAge reached 0.747 and 0.738, respectively. These results were consistent across different age groups and cause‐specific mortality rates (Table S11, Supporting Information). Notably, among young adults (<45 years), ProtAge had a C‐index of 0.793 in survival analysis, highlighting its effectiveness in predicting the risk of premature mortality (Figure 3C). For cause‐specific mortality, ProtAge had a C‐index of 0.754 for cancer mortality and 0.850 for heart disease mortality, the highest among the three ageing clocks. Compared with those with MetAgeDiff and BioAgeDiff, individuals in the top 25% according to ProtAgeDiff presented the highest cumulative mortality incidence rates throughout the follow‐up period (Figure S5, Supporting Information). Pathway enrichment analysis of ProtAge proteins (Figure S5, Supporting Information) identified regulation of MAPK cascade (HGF, GDF15, EGFR, etc.), and regulation of intracellular signal transduction (REN, AGER, KIT, ect.), highlighting their potential roles in regulating cellular senescence traits.^[^ ^27^ ^]^

We then decomposed ProtAgeDiff into contributions from cardiometabolic (CardioDiff), inflammatory (InflamDiff), neurological (NeuroDiff), and oncological (OncoDiff) proteins (Figure 3D,E), which may reveal various aspects of ageing mechanisms. CardioDiff and NeuroDiff emerged as the top two contributors to ProtAgeDiff and demonstrated the highest C‐index in survival analysis (Figure 3F; Figures S6,S7, Supporting Information). Among these proteins (Table 2), GDF15, NTproBNP, and EGFR have been identified as ageing biomarkers, and NEFL is frequently highlighted among neurological proteins. Given the relative independence of the four ProtAgeDiff categories (Figure 3G), we used the counts within the high‐risk group (top 25% of Cardio/Neuro/Inflamm/Onco Diff) to produce a risk score ranging from 0 to 4. This risk score effectively identified individuals at high risk of mortality (Figure 3H). For example, approximately 60% of people who scored 4 died within approximately 16 years due to all‐cause mortality. In summary, ProtAge and its ProtAgeDiff serve as omics‐based ageing clocks for predicting mortality risk, and the ProtAgeDiff calculation allows us to analyse the ageing process across four distinct biological categories.

### 2.4 GOLD BioAge and Incident Chronic Diseases

**Figure 4:**
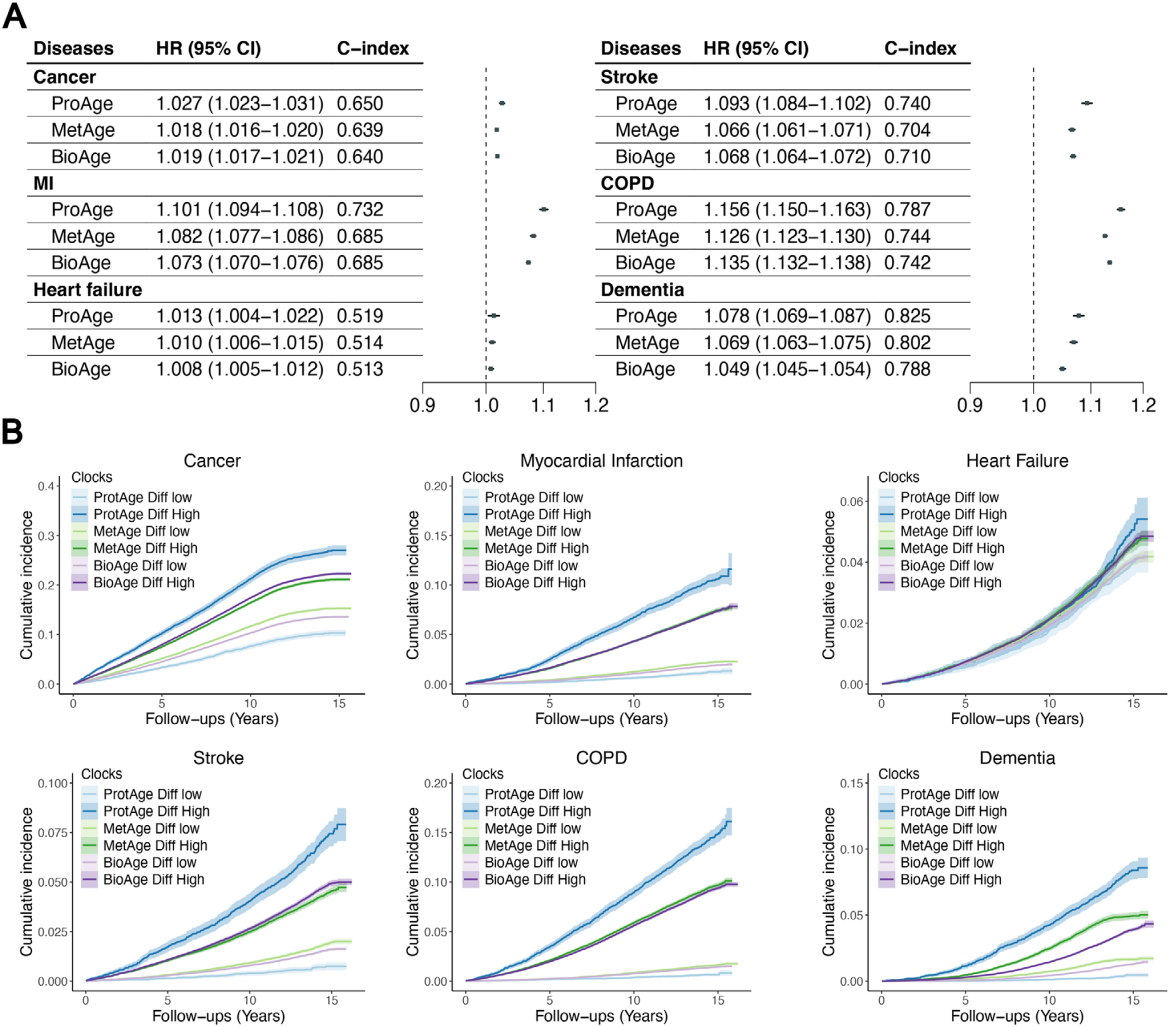
Associations between GOLD ProtAge, MetAge, and BioAge and the incidence of age‐related chronic diseases. The forest plots A) illustrates the hazard ratios and C‐index for ProtAge, MetAge, and BioAge across chronic diseases in the UKB. These associations were adjusted for age and sex. MI: myocardial ischemia; COPD: chronic obstructive pulmonary disease; CI: confidence interval. Survival plots are displayed based on the differences between ProtAge, MetAge, and BioAge relative to chronological age, referred to as ProtAgeDiff, MetAgeDiff, and BioAgeDiff. The high and low risk groups correspond to the top and bottom 25% of the population, respectively.

To investigate the potential of GOLD BioAge to predict the incidence of common chronic diseases, we included cancer, myocardial infarction (MI), heart failure, stroke, chronic obstructive pulmonary disease (COPD), and dementia in the association analysis. The Cox proportional hazards model showed that a 1‐year increase in biological age was associated with an increased risk of disease (**Figure** **4**A). For example, in the case of cancer, a 1‐year increase in ProtAge, MetAge and BioAge was associated with increases of 2.7%, 1.8%, and 1.9% in hazard ratios (HRs), respectively. This trend was consistent across other diseases, such as myocardial infarction and stroke. Moreover, the ProtAge model demonstrated slightly higher HRs and C‐index values for most specific diseases than the BioAge model did. For dementia, for example, the HR of ProtAge reached 1.078 (1.069–1.087) per 1‐year increase, whereas BioAge had an HR of 1.049 (1.045–1.054). Similarly, the MetAge model exhibited robust performance across diseases such as MI and stroke, with HRs of 1.082 (1.077–1.086) and 1.066 (1.061–1.071), respectively. These results highlight the value of ProtAge and MetAge in predicting incident chronic diseases in large cohorts.

Cumulative disease incidence trajectories are presented on the basis of the pace of ageing as measured by ProtAgeDiff, MetAgeDiff, and BioAgeDiff (Figure 4B). The differences between the highest and lowest ProtAgeDiff groups were most pronounced among the three metrics, indicating that ProtAge was particularly effective in predicting the onset of chronic diseases. Over a follow‐up period of 16 years, the cumulative incidences for cancer, myocardial infarction, heart failure, stroke, chronic obstructive pulmonary disease (COPD), and dementia in the high‐ProtAgeDiff group were 27.0%, 11.6%, 5.4%, 7.9%, 16.1%, and 8.6%, respectively. Overall, these findings underscore the potential of ProtAge and MetAge to predict age‐related chronic diseases.

### 2.5 Comparison with Other Ageing Clocks

To investigate the validity of our models, we compared the mortality prediction performance of the GOLD BioAge model with Levine's phenotypic age, KDM biological age (KDM‐BA), and chronological age in the NHANES (8,106 participants, aged 47.0 ± 16.3 years) and UKB (265,541 participants, aged 56.5 ± 8.0 years). These ageing clock models were constructed using clinical biomarkers with chronological age included as a reference.

**Figure 5:**
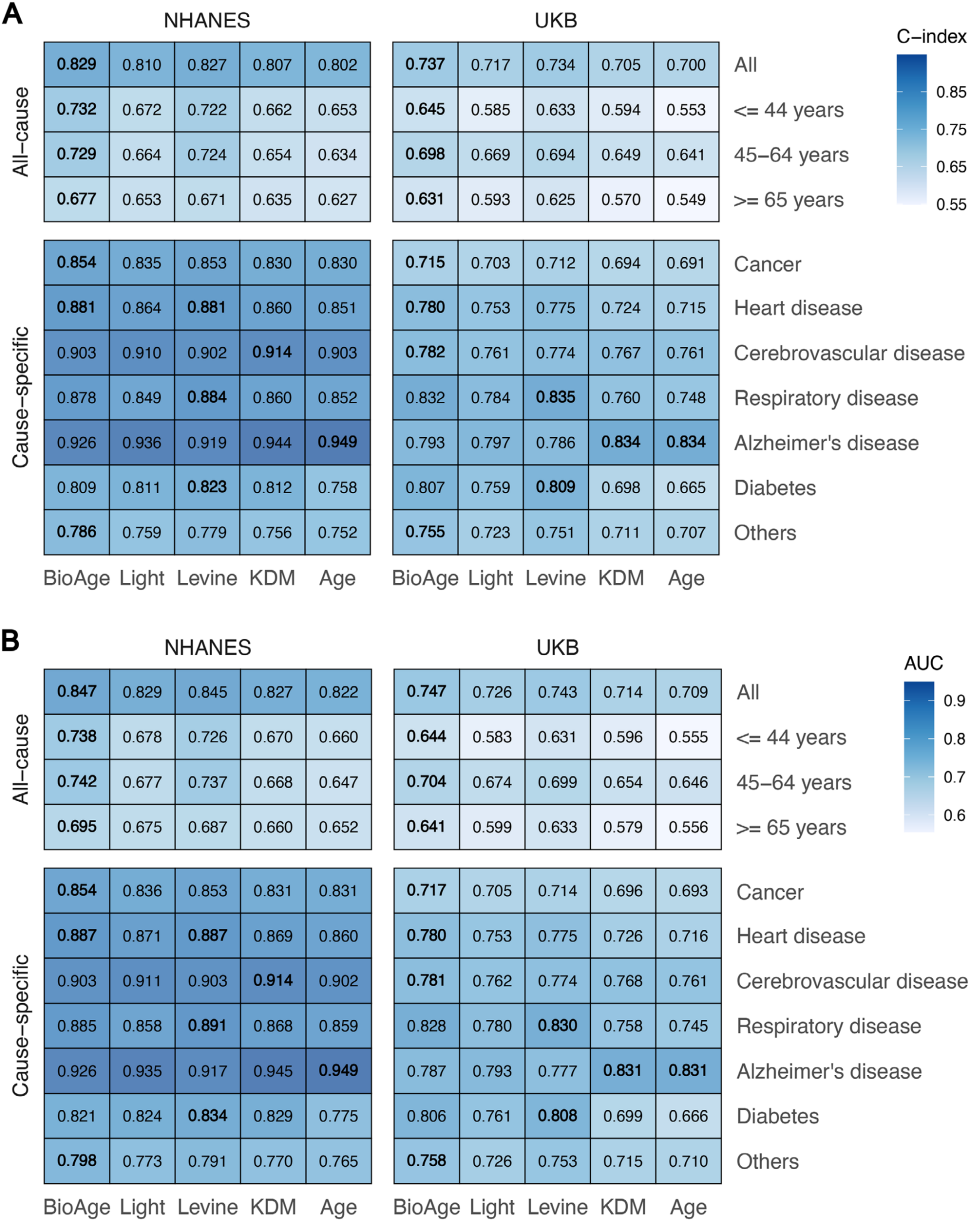
Comparison of GOLD BioAge and other common aging clocks in predicting mortality in NHANES and UKB. The C‐index in survival analysis A) and AUC value of 10‐year mortality prediction B) of these aging clocks are shown. Both all‐cause (age‐stratified) and cause‐specific mortality are considered. The highest value is marked with bold. The BioAge, Light, Levine and KDM referred to the GOLD BioAge, its light version, Levine's phenotypic age, KDM algorithm derived age, respectively.

**Figure** **5** shows the C‐index of survival analysis and AUC values for 10‐year mortality prediction of these ageing clocks. The BioAge model performed significantly better overall than other biological and chronological age models across the NHANES and UKB datasets, both in the overall samples and within specific age groups. For example, the BioAge model achieved a C‐index of 0.829 in the NHANES, outperforming Levine's phenotypic age (0.827), KDM (0.807), and chronological age (0.802). For cause‐specific mortality, the GOLD BioAge model had the highest C‐index and AUC values among these ageing clocks. Taking mortality related to heart disease as an example, the C‐index of the BioAge was 0.881 in the NHANES and 0.780 in the UKB. We used the NHANES III dataset as the validation dataset (Figure S8, Supporting Information), and the GOLD BioAge showed competitive performance compared with these common ageing clocks. These results confirmed the validity of our biological age algorithm and its ability to capture mortality risk.

### 2.6 Light BioAge for Practical Simplicity

For simplicity in clinical practice, we refined and simplified GOLD BioAge as a light version called Light BioAge (Figure S9, Supporting Information). The Light BioAge model incorporates chronological age, serum creatinine, glucose, and log‐transformed C‐reactive protein (Log CRP). The calculation formula is as follows:

(3) $$\begin{array}{ccc} Light\;BioAge & = & Age+8.3313*Creatinine+0.8270*Glucose\\ & & +\;5.7305\;*\;Log\;CRP-13.5298 \end{array}$$

**Figure 6:**
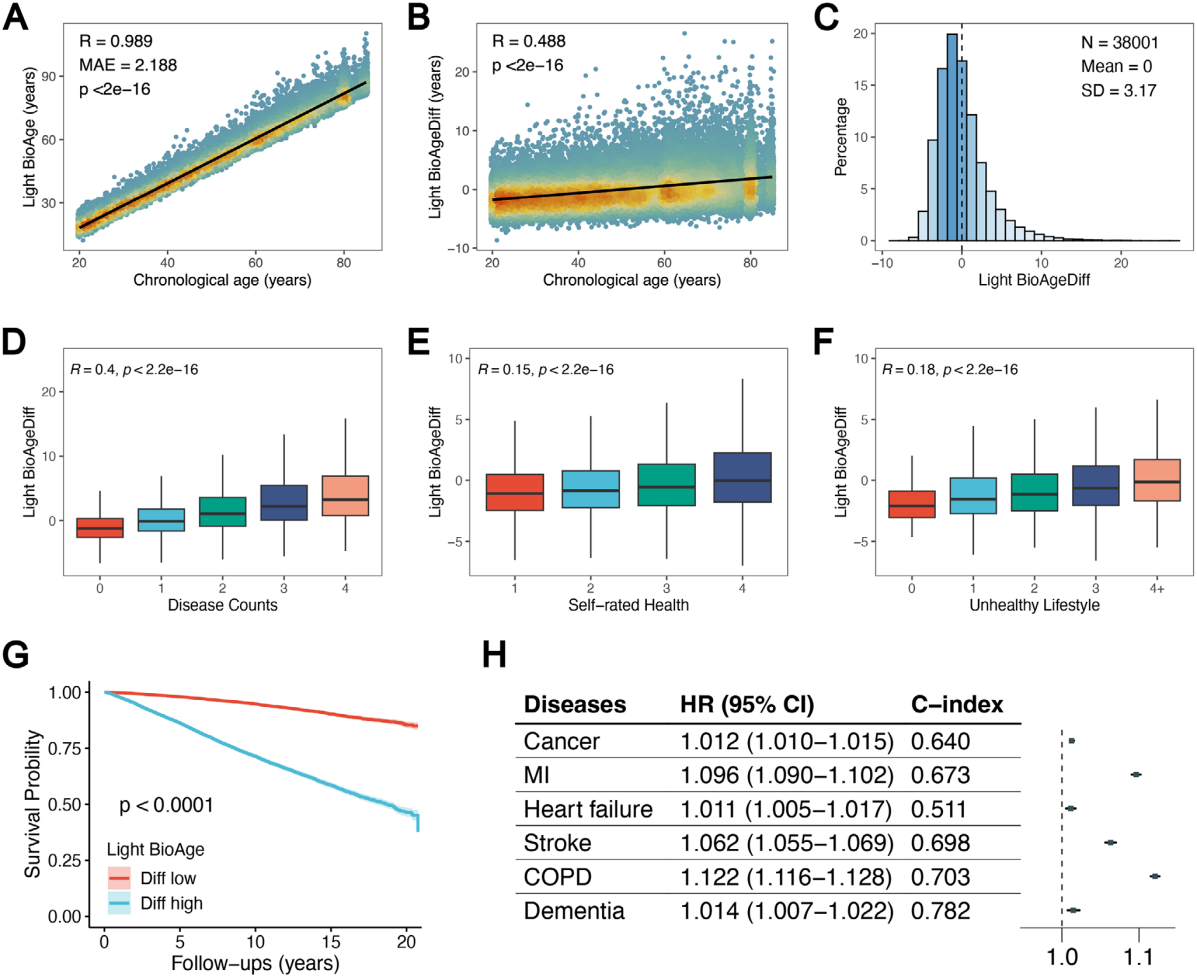
The Light BioAge and its associations with age‐related factors and outcomes. The correlation of Light BioAge with age A). And the difference of Light BioAge with age also correlated with age B) and its distribution C). The correlations of Light BioAgeDiff with counts of age‐related chronic diseases D), self‐rated health E), and unhealthy lifestyles F). The survival plot G) based on Light BioAgeDiff levels, with the top and bottom 25% of the population representing the high and low risk groups. The forest plots H) show the hazard ratios and C‐index of Light BioAge in relation to chronic diseases, adjusted for age and sex. MI: myocardial ischemia; COPD: chronic obstructive pulmonary disease; HR: hazard ratio; CI: confidence interval.

In the NHANES, 38,001 samples (49.6 ± 18.3 years old) with these three biomarkers were included. Light BioAge was strongly correlated with chronological age (R = 0.989, **Figure** **6**A), which accounted for 93.73% of the variance in the GOLD BioAge. Its difference from chronological age (Light BioAgeDiff) was positively correlated with age (Figure 6B), which followed a nearly normal distribution (Figure 6C). It was also significantly associated with comorbidities, self‐rated health, unhealthy lifestyles, and the risk of mortality (Figure 6D–G, Table 1).

Compared with the GOLD BioAge model, the Light BioAge model, which utilizes the fewest indicators, demonstrated competitive predictive accuracy (Figure 5). In the NHANES dataset, while the full BioAge model achieved a higher C‐index of 0.829 for all‐cause mortality, the light model demonstrated competitive performance with a C‐index of 0.810. Furthermore, the C‐index of Light BioAge was very close to previous prominent measures, such as Levine's phenotypic age and KDM. For example, for mortality from cerebrovascular disease, the C‐index of Light BioAge reached 0.911, which is comparable to the phenotypic age (0.903) and KDM (0.914) reported in the NHANES. Notably, to enhance its clinical applicability, we evaluated its performance in predicting incident chronic disease. For example, the Light BioAge model demonstrated HRs of 1.122 (1.116–1.128), 1.096 (1.090–1.102), and 1.062 (1.055–1.069) for COPD, MI, and stroke, respectively (Figure 6H). These results indicate that Light BioAge provides a robust and practical alternative while remaining competitive with other ageing metrics.

### 2.7 Light BioAge Predicted Mortality in Validation Cohorts

We further validated Light BioAge in three independent datasets, the CHARLS (17,163 participants, aged 58.4 ± 10.05 years), RuLAS (1,785 participants, aged 77.0 ± 4.2 years), and CLHLS (2,499 participants, aged 85.5 ± 12.0 years) datasets. In the three cohorts (Table 1), 1752, 186, and 813 deaths occurred during the median follow‐up periods of 9.0, 4.0, and 4.1 years, respectively.

**Figure 7:**
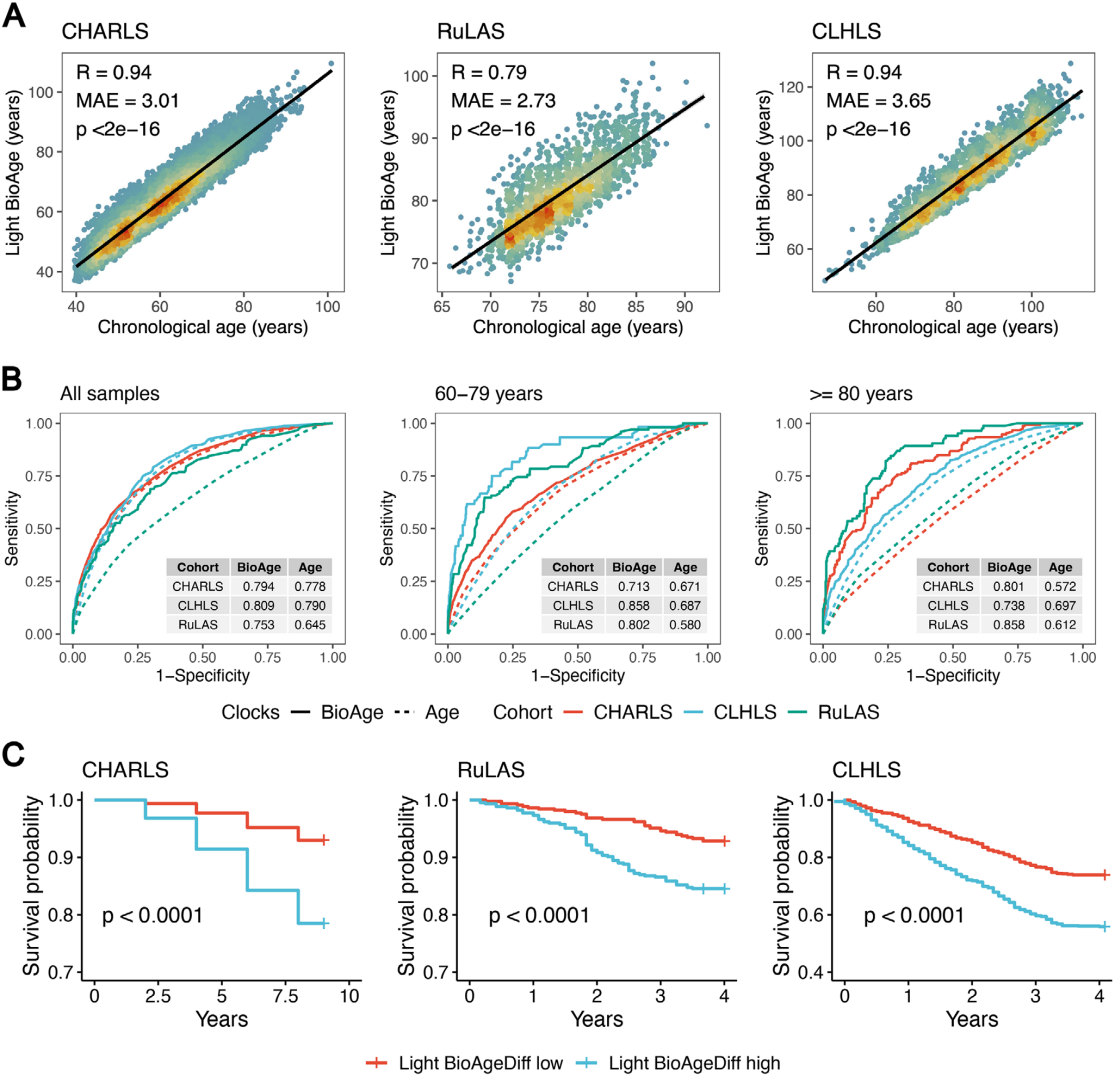
Validations of the Light BioAge in three independent cohorts. The correlations A) of Light BioAge with age in CHALS, RuLAS and CLHLS. The ROC curves B) of Light BioAge (solid lines) and age (dotted lines) for predicting mortality across all samples, and within age‐stratified groups (<80, ≥80 years old). Survival plots C) depict mortality trajectories of individuals categorized based on Light BioAgeDiff levels, with the top and bottom 25% represented as high and low risk groups in CHARLS, RuLAS, and CLHLS.

Light BioAge was strongly correlated with chronological age across the three cohorts (**Figure** **7**A). In the full samples, Light BioAge achieved AUC values of 0.794 in the CHARLS, 0.809 in the CLHLS, and 0.753 in the RuLAS (Figure 7B). These values were greater than those for chronological age, which were 0.778, 0.790, and 0.645, respectively. Notably, Light BioAge outperformed chronological age for individuals aged 60–79 years, with an AUC that exceeded 0.790 in both the CLHLS and RuLAS. It also maintained an approximately robust AUC of 0.8 for those aged 80 and older, significantly outperforming chronological age. Participants with high BioAgeDiff (top 25%) experienced a more pronounced decline in survival probability than did those with low BioAgeDiff (bottom 25%) across the CHARLS, RLAS, and CLHLS (Figure 7C). By the end of the follow‐up periods in each cohort, the survival probabilities of individuals in the high‐risk groups were approximately 78.5.%, 84.5%, and 55.8%, respectively.

**Figure 8:**
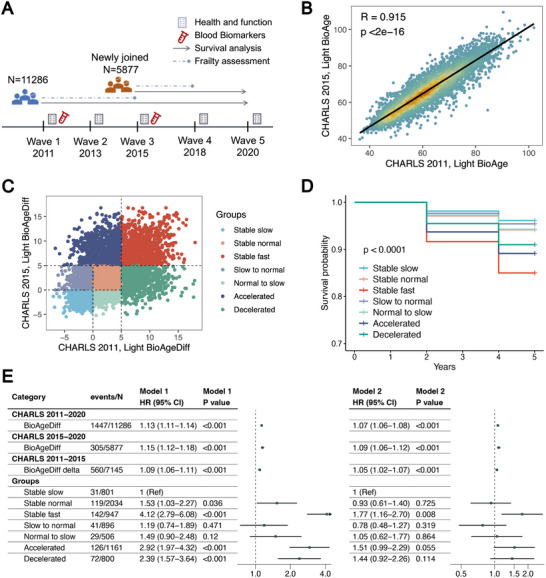
The Light BioAge, its dynamics and mortality in CHARLS. Illustration A) detailing the study designs across five waves in CHARLS. Correlation B) between Light BioAge values in wave 1 (2011) and wave 3 (2015). Scatter plot C) displays Light BioAgeDiff in wave 1 (2011) and wave 3 (2015), with dotted lines indicating Light BioAgeDiff values of 0 and 5. Individuals were divided into 7 groups based on the changes in Light BioAgeDiff, with survival plots D) and forest plots E) provided. Model 1 represented the crude model, while Model 2 adjusted for age and sex.

Considering human ageing as a longitudinal process, we examined the dynamic changes in Light BioAgeDiff between wave 1 and wave 3 of the CHARLS (**Figure** **8**A). Light BioAge in the two waves was strongly correlated (R = 0.915, Figure 8B), whereas the Light BioAgeDiff showed a moderate correlation (R = 0.475). In accordance with the Light BioAgeDiff, participants were classified into slow (Diff < 0), normal (0 ≤ Diff < 5) and fast (Diff > 5) ageing groups, which were subsequently classified into seven categories on the basis of their ageing status across both waves (Figure 8C). The stable slow‐ageing groups across the two waves were used as the reference. Compared with the reference group, the stable fast‐aging and accelerated aging groups (slow/normal to fast) presented the highest mortality risk (Figure 8D,E). In addition, the decelerated ageing group (fast to slow/normal) was associated with a reduced risk of mortality.

### 2.8 Light BioAgeDiff, Frailty and Mortality Risks

**Figure 9:**
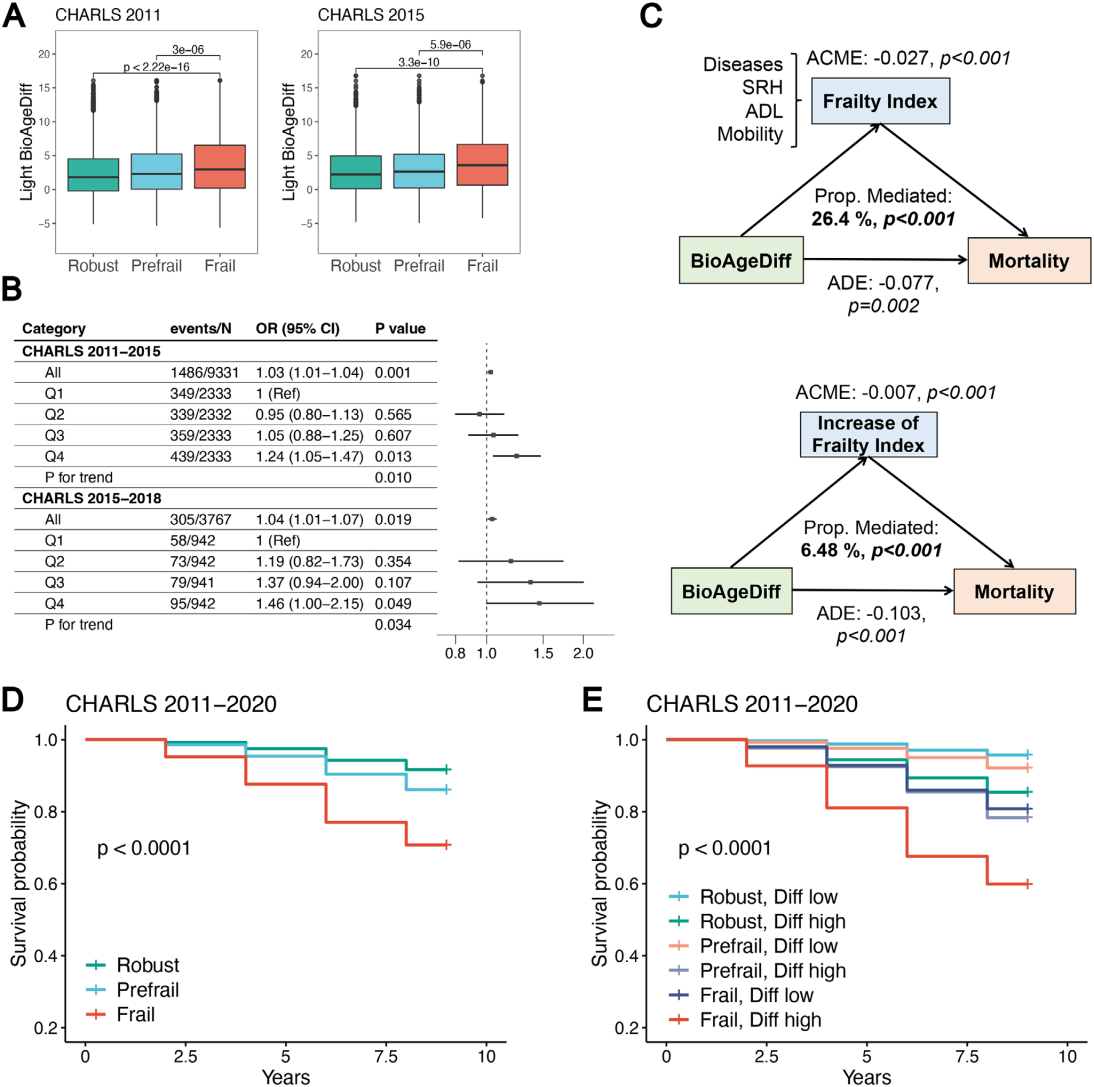
The Light BioAgeDiff, frailty and mortality in CHARLS. The boxplots A) of Light BioAgeDiff across robust, prefrail, and frail individuals in CHARLS waves 1 (2011) and 3 (2015), with statistical significance determined using Wilcoxon tests. Forest plots B) illustrates the associations between Light BioAgeDiff and incidence of frailty. The odd ratios were calculated through continuous and category Light BioAgeDiff (Q1‐4: quartiles), adjusted by age and sex. The mediation models C) of Light BioAge (wave 1, 2011), frailty index (wave 3, 2015) and mortality (wave 3–5, 2015–2020). The change in frailty index was calculated based on assessments from waves 1 and 3. SRH: self‐rated health; ADL: activities of daily living; ADE: average direct effect; ACME: average causal mediated effect. The survival plots of individuals according to frailty status D), and both frailty status and levels of Light BioAgeDiff E).

Next, we explored the associations of Light BioAgeDiff with frailty as assessed by the frailty index, which included age‐related chronic diseases, self‐rated health, basic and instrumental activities of daily living and mobility capacity. In the CHARLS 2011 and 2015, frailty status was associated with BioAgeDiff, in which frail individuals were 1.14 and 1.20 years older, respectively, than their robust counterparts (**Figure** **9**A). During longitudinal follow‐up (2011‐2015, 2015–2018), the baseline BioAgeDiff was associated with incident frailty (odds ratio [95% CI]: 1.03 [1.01‐1.04]; 1.04 [1.01‐1.07], Figure 9B). The participants in the fourth quartile of BioAgeDiff presented the highest risk. Using BioAgeDiff as a measure of biological ageing, we examined the mediating role of functional decline, measured by the frailty index, in the associations of BioAgeDiff with mortality risk (Figure 9C). The frailty index mediated approximately 26.4% (*p* < 0.001) of the relationship with an increase accounting for 6.48% of the variance.

Light BioAgeDiff exhibited a performance similar to that of frailty in predicting mortality, with C‐index values of 0.634 for BioAgeDiff and 0.633 for frailty in the CHARLS study. We used BioAgeDiff and frailty as measures of biological and functional ageing and examined their combined effectiveness in identifying individuals at high risk of mortality. The mortality rate of frail individuals was approximately 29.2% during the 9‐year follow‐up according to the CHARLS (Figure 9D). In contrast, frail individuals with the highest BioAgeDiff had a mortality rate of approximately 40.1% during this period (Figure 9E). These findings highlight the potential role of Light BioAgeDiff in preventing incident frailty and its joint contribution with frailty in identifying individuals at elevated risk for mortality.

## 3 Discussion

In this study, we introduced a robust algorithm to estimate biological age as a linear combination of chronological age and various biomarkers. GOLD biological age and its difference from chronological age provide insights into the relationship between individual biomarker values and the pace of ageing. Notably, the implementation of our algorithm in proteomics and metabolomics demonstrated the significant potential of omics biomarkers in identifying the risks of mortality and age‐related chronic diseases. Furthermore, benchmark analysis demonstrated that our models outperformed traditional ageing clocks in predicting the risks of both all‐cause and cause‐specific mortality across different age groups. We also developed a simplified version called Light BioAge, which provides a practical and efficient alternative with simplified calculations. Light BioAge exhibited predictive capabilities in assessing mortality risk across three validation cohorts of elderly participants and was associated with the onset of frailty. In summary, our algorithm was validated as a general framework for constructing ageing clocks. Importantly, both GOLD BioAge and its light version can serve as convenient tools for ageing assessment in clinical practice.

The robustness of the GOLD BioAge algorithm and the ageing clocks was validated through multiple aspects. The evaluation of GOLD BioAge focused primarily on the correlation between BioAgeDiff and chronological age as well as its ability to predict all‐cause and cause‐specific mortality, the incidence of multiple age‐related chronic diseases, the onset of frailty, and validations across diverse populations. Benchmark analyses of mortality prediction demonstrated the superiority and sensitivity of the GOLD BioAge model. In summary, the GOLD BioAge serves as a general measure of biological ageing and offers simple and practical calculations for ageing assessment and public health.

The pace of individual ageing undergoes dynamic changes throughout life and is influenced by modifiable lifestyles, environmental factors, psychological influences, and health conditions. Identifying individuals at high risk of premature ageing can increase primary prevention efforts and reduce the health care and socioeconomic burdens linked to age‐related diseases. In this study, GOLD BioAge estimated individuals’ biological ageing status and captured the risks of morbidity and mortality. To further promote the application of biological age in public health and clinical settings, we introduced Light BioAge, a simple and practical ageing clock that utilizes only three accessible biomarkers in addition to chronological age. Light BioAge demonstrated applicability across various independent cohorts (NHANES, UKB, CHARLS, CLHLS, and RuLAS) with diverse study designs, participant characteristics, and morbidity profiles. This model incorporates serum creatinine, blood glucose, and C‐reactive protein levels with chronological age to reflect kidney function and metabolic and inflammatory status. These biomarkers are commonly used in medical examinations and are easily accessible at low cost. Therefore, Light BioAge offers a convenient tool for ongoing monitoring of ageing trajectories to prevent functional decline and age‐related diseases.

Compared with Levine's phenotypic age, we estimated biological age by fitting Gompertz mortality hazard function to empirical mortality data. Levine's phenotypic age has been widely used in ageing‐related studies. Notably, phenotypic age outperformed earlier biological age methods in predicting all‐cause mortality and various diseases.^[^ ^28^ ^]^ GOLD BioAge exhibited a strong correlation with Levine's phenotypic age in the NHANES and UKB datasets, confirming the robustness and reliability of our algorithm. Notably, Levine's phenotypic age relies on the Gompertz cumulative distribution function to estimate the 10‐year mortality risk. Its calculation involves a double logarithmic transformation, which complicates its clinical interpretation. In comparison, the calculation of GOLD BioAge is simplified by employing the hazard function to identify the instantaneous mortality risk.

In addition to ageing clocks based on clinical biomarkers, our study introduced ProtAge and MetAge, which incorporated omics data into the GOLD biological age framework. These ageing clocks generally outperform clinical marker‐based clocks in predicting mortality, which may be due to the higher sensitivity and coverage of omics data in capturing ageing‐related information.^[^ ^29^ ^]^ For ProtAge, proteins are categorized into four groups on the basis of their physiological function, with each group contributing to a distinct age estimate. Protein expression and posttranslational modifications, particularly those linked to inflammation, oxidative stress, and cell cycle regulation, provide stable, long‐term biomarkers for clinical outcomes. Additionally, because protein alterations often precede the onset of chronic diseases, proteomics enhances early disease detection, making ProtAge a valuable tool for predicting mortality and early‐stage health risks.^30^, ^31^, ^32^ ^]^ Metabolomics reflects rapid, short‐term fluctuations in the body's biochemical processes, which offers insights into the effect of recent changes in diet, physical activity, and stress on ageing. By integrating both proteomic and metabolomic data into the ageing clock, we created a more comprehensive tool for estimating biological age. The GOLD omics ageing clocks provide the potential for personalized health interventions to mitigate ageing‐related risks.

Organ‐specific ageing clocks,^[^^]^ such as brain age measured through magnetic resonance imaging (MRI) data^[^ ^34^, ^35^ ^]^ have been developed to evaluate biological ageing in individual organs. Proteomic data further contribute to this understanding by including organ‐specific proteins, which predict chronic diseases linked to those organs.^[^ ^14^, ^36^ ^]^ However, research reveals that ageing trajectories differ markedly across organs, with distinct patterns that deviate from one another and from the body's overall rate of ageing.^[^ ^33^, ^37^ ^]^ These observations emphasize the need to enhance the GOLD BioAge framework to address organ‐level variations in ageing and better capture these biological disparities in the future.

This study has several limitations. First, although omics‐based ageing clocks demonstrated superior performance compared with those that used clinical biomarkers in the UKB dataset, further validation in other elderly cohorts is essential to confirm these findings. Additionally, biomarkers for ageing clocks were selected via LASSO penalized regression to increase accuracy; however, different feature selection methods could yield alternative sets of biomarkers, indicating the potential for further optimization of biomarker panels in clinical applications. While Light BioAge and GOLD BioAge demonstrated potential as practical clinical tools, ProtAge and MetAge, as advanced research‐oriented models, lacked immediate clinical applicability due to their technical complexity and reliance on specialized data. Furthermore, we validated Light BioAge in three Chinese cohorts, but it is uncertain whether the full GOLD BioAge model would more accurately capture the risks associated with geriatric syndromes and mortality.

## 4 Experimental Section

### Study Populations

This study used the data of the NHANES 1999–2018, UKB, CHARLS, CLHLS, and RuLAS, with baseline participant characteristics summarized in Tables S1,S2 (Supporting Information). The US NHANES was a nationally representative cross‐sectional survey of civilians living in the US that was approved by the National Center for Health Statistics (NCHS) Ethics Review Board.^[^ ^38^ ^]^ The UK Biobank was a large‐scale prospective cohort that collects data from over 500,000 participants across 22 centres in England, Scotland, and Wales. The UKB received ethics approval from the North West Multicentre Research Ethics Committee.^[^ ^39^ ^]^ The CHARLS was an ongoing population‐based longitudinal cohort study of middle‐aged and older Chinese adults. It was approved by the Ethics Review Board of Peking University in accordance with the Declaration of Helsinki and other relevant guidelines and regulations.^[^ ^40^ ^]^ The CLHLS was a nationwide longitudinal study of the elderly Chinese population. The project was approved by the Biomedical Ethics Committee of Peking University, China (IRB00001052‐13074).^[^ ^41^ ^]^ The Rugao Longevity and Ageing Study (RuLAS) was a population‐based prospective study in Rugao, China, that consists of a longevity cohort and an ageing cohort.^[^ ^42^ ^]^ The RuLAS was approved by the Human Ethics Committee of Fudan University School of Life Sciences. All participants provided written informed consent. This study followed the Strengthening the Reporting of Observational Studies in Epidemiology (STROBE) reporting guidelines for cohort studies.^[^ ^43^ ^]^

### Clinical Biomarker Selection for Constructing GOLD BioAge

Data was utilized from the NHANES 1999–2018 for variable selection and refined the biomarker panel to construct the GOLD biological age. A total of 26 common biomarkers from cell blood count (CBC) tests and biochemical assays were included (Table S3, Supporting Information). LASSO‐Cox regression models were employed to select biomarkers for predicting all‐cause mortality, with fivefold cross‐validation to determine the optimal lambda value (lambda = 0.0166). Among the initial 26 biomarkers, 9 biomarkers were retained (Figure S1, Supporting Information). This set of biomarkers formed the basis for the biological age model (GOLD BioAge). To make the panel more practical for use, feature selection was conducted on a set of 10 blood (biochemical and haematological) biomarkers that were consistently collected across the aforementioned five cohorts (Table S4, Supporting Information). This simplified panel, including chronological age, serum creatinine, glucose, and C‐reactive protein (CRP), formed the Light GOLD Biological Age Model (Light BioAge).

### Metabolomics and Proteomics Biomarker Selection

GOLD BioAge models were applied to metabolomic and proteomic biomarkers, employing data from the UK Biobank (UKB, 2006–2010). For the GOLD Proteomic Age Model (GOLD ProtAge), 2,923 proteins from 53,014 participants were analyzed. For quality control, proteins were excluded with more than 10% missing data and removed participants with more than 50% missing proteins, resulting in 1,459 protein biomarkers for feature selection. A LASSO‐Cox regression model was then used with fivefold cross‐validation optimized for all‐cause mortality prediction. To optimize both model performance and simplicity, for a lambda value (model penalty) of exp (‐4) was opted, which selected fewer features while maintaining a relatively high C‐index (Figure S1, Supporting Information). Finally, 22 protein biomarkers associated with chronological age were selected. The final dataset included 39,772 participants for downstream analysis, after excluding participants with missing data of these 22 proteins (Table S5, Supporting Information). For the metabolomics data in UKB, a total of 248,202 UKB participants were enrolled, each with measurements of 251 circulating metabolomic markers. Similar to protein feature selection, LASSO Cox regression was conducted for all‐cause mortality via fivefold cross‐validation. Consequently, 26 metabolomic biomarkers were selected on the basis of the lambda value of exp (‐6), which corresponded to an increase of one standard deviation over the lambda value with maximum C‐index. Detailed descriptions of all selected variables were available in the supporting Information. The robustness of Lasso‐Cox regression‐derived biomarkers was systematically assessed through stability testing against data perturbations and subsampling strategies (Supporting Information). The sensitivity analyses demonstrated consistent selection of biomarkers, confirming their robustness to perturbations and subsampling variability.

### GOLD BioAge Model Training

Two Gompertz regression models were conducted for biological age model training. The first Gompertz regression model included only chronological age as a predictor of time‐to‐mortality data. The second Gompertz regression model incorporated both chronological age and selected biomarkers as predictors. GOLD biological age (GOLD BioAge) was defined as age accounting for the actual mortality hazard by considering both chronological age (CA) and additional biomarkers (Figure S2, Supporting Information). The models were specified as follows:

Model 1: chronological age only:

(4) $$h_{1}\left(t\right)=rate_{1}\;*\;exp\left(\beta_{1}\;*\;CA+shape_{1}\;*\;t\right)$$

Model 2: chronological age and selected biomarkers:

(5) $$h_{2}\left(t\right)=rate_{2}*\exp\left(\beta_{2}\;*\;CA+\sum \beta_{2i}\;*\;Biomarker_{i}+shape_{2}\;*\;t\right)$$

In Model 2, biomarkers represented the selected biomarkers included in the model, where *Biomarker_i_* was the i‐th biomarker and β_2*i*_ represented the coefficient of each biomarker in model 2. GOLD BioAge integrates chronological age with relevant biomarkers to better estimate mortality hazard and ageing status. Let *h*_1_(*Bioage*, *t*  =  0) ≈ *h*_2_(*CA*,*Biomarkers*, *t*  =  0). In the real dataset, the empirical values of *h*_1_ and *h*_2_ were slightly different (Figure S2, Supporting Information) but strongly correlated (Pearson r = 0.969). To reduce estimation bias for biological age in the whole population, a variable (γ) was added to correct bias and let *h*_1_ = γ*h_2_  and $\gamma =\frac{h_{1}}{h_{2}}\;$.

Thus, GOLD BioAge was derived as follows:

(6) $$GOLD\;BioAge=\frac{1}{\beta_{1}}\left(\beta_{2}*CA+\sum \beta_{2i}\;*\;Biomarker_{i}+\log\frac{\gamma \;*\;rate2}{rate1}\right)$$

In the equation, log denoted the natural logarithm. For further simplify the formula of GOLD BioAge, the coefficient parameter of CA (β_2 _) was set equal to the parameter (β_1_) in Model 2, and estimated the parameters for the rate, shape, and coefficients of biomarkers in Model 2. When β_2 _ = β_1 _ , the formula was further simplified as follows:

(7) $$GOLDBioAge=CA+\sum \frac{\beta_{2i}}{\beta_{1}}\;*\;Biomarker_{i}+\frac{1}{\beta_{1}}*\log\frac{\gamma \;*\;rate2}{rate1}$$

The Gompertz distribution parameters (rate, shape, and coefficients) were estimated by maximum likelihood using the “flexsurv” R package. The constant item, $\frac{1}{\beta_{1}}*\log\frac{\gamma *rate2}{rate1}$, was estimated by the mean value of $\frac{1}{\beta_{1}}*\log\frac{\frac{h_{1}}{h_{2}}*rate2}{rate1}$, where *h*_1_ and *h*_2_ were the empirical values of hazard risk for the two Gompertz models. The detailed coefficients of GOLD BioAge, Light BioAge, ProtAge and MetAge were shown in Tables S6–S9 (Supporting Information). This methodological validation included direct comparison of Gompertz versus Cox regression frameworks for biological age derivation (Supplementary Methods). The biomarker coefficients exhibited high concordance between approaches, driving perfect alignment (R = 1.00) between GOLD BioAge and Cox BioAge constructs (Figure S10, Supporting Information). The algorithm of GOLD BioAge was implemented as an R package (http://github.com/Jerryhaom/GOLDBioAge). The GOLD BioAge tools were also accessible through an online web calculator (https://jerryhaom.github.io/GOLDBioAgeWeb.io/).

### Benchmark of Biological Age Models

To ensure the robustness of the models, their performance in the NHANES and UKB was assessed compared with other established phenotypic ageing clocks and dimensional reduction methods. Levine's phenotypic age, KDM age and the Mahalanobis distance statistic were calculated using the “BioAge” R package.^[^ ^44^ ^]^ PCA age was calculated through principal component analysis with the first five components regressed to age. For benchmarking analyses, NHANES and UK Biobank (UKB) samples were restricted to participants with complete biomarker profiles required for the aging clock models. Predictive performance was assessed through survival model discrimination (C‐index) and binary classification accuracy (AUC) for 10‐year mortality, with stratified analyses conducted across: the full cohort, age‐stratified subgroups: young adults (<45 years), middle‐aged adults (45–64 years), and older adults (>65 years), and sex‐specific subgroups (Tables S10,S11, Supporting Information). Additionally, the ability of these ageing clocks were examined to predict cause‐specific mortality. Since each ageing clock served as a single variable, logistic regression models were used to estimate 10‐year survival predictions solely on the basis of these ageing clocks and age. The C‐index of survival analysis (crude model) was used to evaluate the discriminatory power of these ageing clocks in predicting all‐cause and cause‐specific mortality. This comprehensive benchmarking analysis provided a thorough evaluation of the models' performance and facilitated comparisons with other established ageing clocks.

### Assessment of Mortality and Onset of Chronic Diseases

In the NHANES, death information was based on linked data from records taken from the National Death Index (NDI) through December 31, 2019, provided through the Centers for Disease Control and Prevention. Data on mortality status and length of follow‐up (in person‐months) were available for nearly all participants. In the UKB, death information was obtained from death certificates held within the National Health Service (NHS) Information Centre (England and Wales) and the NHS Central Register (Scotland) to November 30, 2022. Participants’ time was calculated to death from baseline to the date of death, date of loss to follow‐up, or date of last record of follow‐up, whichever came first. The International Statistical Classification of Diseases, 10^th^ edition was used, to define causes of death. Cause‐specific mortality included mortality from malignant neoplasms, heart disease, cerebrovascular disease, respiratory disease, Alzheimer's disease, diabetes, and others. In addition, data on the dates of incident chronic disease in the UKB, including cancer, myocardial infarction, heart failure, stroke, chronic obstructive pulmonary disease (COPD), and dementia, were collected. The associations of GOLD BioAge, Light BioAge, ProtAge, and MetAge with mortality and diseases were shown in Tables S12–S15 (Supporting Information).

### Assessment of Health‐Related Factors and Outcomes

The unhealthy lifestyle score was based on six modifiable lifestyle factors, namely, smoking, alcohol consumption, physical activity, diet, body mass index (BMI), and sedentary behavior, as defined by the World Health Organization. The score was categorized into five groups (0, 1, 2, 3, 4 and more unhealthy factors). Multimorbidities were defined as the number of lifetime disease diagnoses. In the NHANES, diabetes, high blood pressure, congestive heart failure, coronary heart disease, heart attack, stroke, cancer or malignancy, and chronic bronchitis were included. In the UKB, cancer, myocardial infarction, heart failure, stroke, chronic obstructive pulmonary disease (COPD), and dementia was included. Disease count was classified into five categories: no disease and 1, 2, 3, and 4 or more diseases. Self‐rated health was recorded on four levels: excellent or very good, good, fair and poor. The distributions of GOLD BioAge, Light BioAge, ProtAge, and MetAge by unhealthy lifestyle, comorbidity, and self‐rated health were shown in Table S16 (Supporting Information).

### Validation in Independent Elderly Cohorts

The Light BioAge model was validated in three additional elderly cohorts: the CHARLS, RuLAS and CLHLS datasets (Table S2, Supporting Information). Five waves of CHARLS data (2011–2020) were utilized, and blood‐based bioassay data (CHARLS 2011, 2015) were used to construct the Light BioAge. Health and function questionnaires were collected for frailty assessment^[^ ^45^ ^]^ (Table S17, Supporting Information). For the RuLAS, wave 2 (2016) was used as the baseline, and blood biomarker data were obtained. The CLHLS 2014–2018 data were used to validate Light BioAge. Using the R package ‘gbm’, we applied gradient boosting models to survival time‐to‐event data to train Light BioAge and age as mortality predictors. ROC curves were calculated to evaluate the prediction performance of Light BioAge and chronological age across all samples as well as subpopulations stratified by age (60‐79 years; ≥ 80 years). Additionally, survival curves were fit for the low‐risk and high‐risk groups on the basis of the Light BioAgeDiff model.

### Statistical Analysis

Survival analysis was conducted in different age groups. Within the same group, participants were classified into quartiles (Table S18, Supporting Information) based on their BioAge Difference (BioAgeDiff), with the top 25% representing individuals at the highest risk of death. Kaplan‐Meier survival curves were then plotted to compare the predicted survival probabilities between the highest and lowest quartiles of the BioAge. Harrell's Concordance Index (C‐index) was applied to quantify the discrimination accuracy of survival models, while the Area Under the Curve (AUC) served as a metric to evaluate predictive performance for 10‐year mortality status within a binary classification framework. Cox proportional hazard models were conducted to assess the associations between different biological aging clocks, mortality and the onset of chronic diseases. The cox regression models were adjusted for sex and chronological age. The pearson correlation coefficient was used to quantify the correlations. The mediation analysis was conducted through R package ‘mediaton’, through the bootstrap approach. All statistical analyses were performed using R version 4.3.3.

## Conflict of Interest

The authors declare no conflict of interest.

## Author Contributions

M.H., H.Z., J.W. contributed equally to this work. M.H., L.Y., H.Z. performed concept and design. M.H., Z.H., S.J. performed acquisition, analysis, or interpretation of data. M.H., J.W., H.Z. draft the manuscript. All authors performed critical revision of the manuscript for important intellectual content. M.H., H.Z., J.W. performed statistical analysis. X.L., S.W., M.W., Y.H., J.W., J.C., Z.B., L.J. performed administrative, technical, or material support. M.H., Y.L., S.J., Z.H., X.W. performed supervision

## Supporting information



Supporting Information

Supplementary Table

Supporting Information

## Data Availability

The data of RulAS are available through reasonable request from the corresponding author. The data from CLHLS are available at https://opendata.pku.edu.cn/dataset.xhtml. The data from CHARLS are available at https://charls.charlsdata.com/pages/data/111/zh‐cn.html. The data from the NHANES are available at www.cdc.gov/nchs/nhis/index.htm, and the data from the UK Biobank are available upon application at www.ukbiobank.ac.uk/register‐apply. This research was conducted using UK Biobank Resource under Application Number 103791.
