# Supplementary material for: Gompertz Law‐Based Biological Age (GOLD BioAge): A Simple and Practical Measurement of Biological Ageing to Capture Morbidity and Mortality Risks
Source: Adv Sci (Weinh). 2025 Jul 2;12(32):e01765. doi: 10.1002/advs.202501765 (PMC12407260; doi:10.1002/advs.202501765)
Supplement: Supplementary file 1 — Supporting Information [file ADVS-12-e01765-s003.pdf]

ADVANCED SCIENCE
Open Access

Supporting Information

for *Adv. Sci.*, DOI 10.1002/advs.202501765

Gompertz Law-Based Biological Age (GOLD BioAge): A Simple and Practical Measurement of Biological Ageing to Capture Morbidity and Mortality Risks

Meng Hao, Hui Zhang, Jingyi Wu, Yaqi Huang, Xiangnan Li, Meijia Wang, Shuming Wang, Jiaofeng Wang, Jie Chen, Zhi jun Bao, Li Jin, Xiaofeng Wang*, Zixin Hu*, Shuai Jiang* and Yi Li**

Supplementary figures of

Meng Hao et al. Gompertz Law-Based Biological Age (GOLD BioAge): A Simple and Practical Measurement of Biological Aging to Capture Morbidity and Mortality Risks.

Figure S1. Feature selection using LASSO-Cox regression and its stability assessment.

Figure S2. Estimate of GOLD BioAge and BioAgeDiff through Gompertz mortality hazard.

Figure S3. Validation of GOLD BioAge in UKB.

Figure S4. GOLD ProtAgeDiff and MetAgeDiff in UKB.

Figure S5. Protein-protein interaction (PPI) network and functional annotation of ProtAge-associated proteins.

Figure S6. Associations of ProtAgeDiff, MetAgeDiff, and BioAgeDiff with mortality in UKB.

Figure S7. The predictive capability of ProtAge and its subpanels regarding mortality.

Figure S8. The comparison of GOLD BioAge, and other common aging clocks in predicting mortality in NHANES III.

Figure S9. The mortality hazard, Light BioAge and Levine's phenotypic age in NHANES.

Figure S10. Comparative analysis of GOLD BioAge and Cox BioAge.

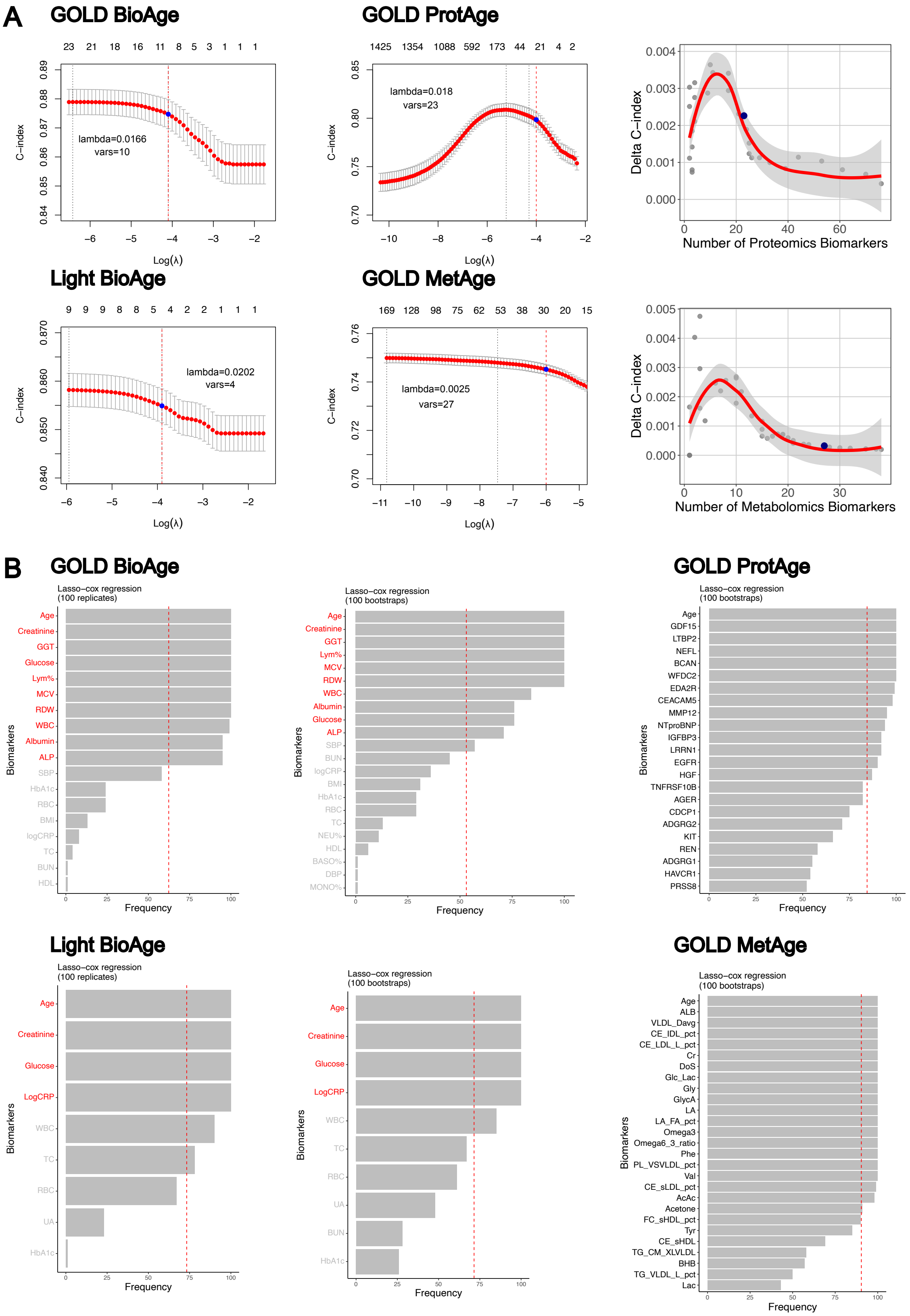

A
GOLD BioAge
lambda=0.0166
vars=10
C−index
Log(λ)
GOLD ProtAge
lambda=0.018
vars=23
Delta C−index
Number of Proteomics Biomarkers
Light BioAge
lambda=0.0202
vars=4
GOLD MetAge
lambda=0.0025
vars=27
Number of Metabolomics Biomarkers
B
GOLD BioAge
Lasso−cox regression
(100 replicates)
Lasso−cox regression
(100 bootstraps)
Biomarkers
Frequency
Age
Creatinine
GGT
Glucose
Lym%
MCV
RDW
WBC
Albumin
ALP
SBP
HbA1c
RBC
BMI
logCRP
TC
BUN
HDL
NEU%
BASO%
DBP
MONO%
GOLD ProtAge
GDF15
LTBP2
NEFL
BCAN
WFDC2
EDA2R
CEACAM5
MMP12
NTproBNP
IGFBP3
LRRN1
EGFR
HGF
TNFRSF10B
AGER
CDCP1
ADGRG2
KIT
REN
ADGRG1
HAVCR1
PRSS8
Light BioAge
LogCRP
UA
GOLD MetAge
ALB
VLDL_Davg
CE_IDL_pct
CE_LDL_L_pct
Cr
DoS
Glc_Lac
Gly
GlycA
LA
LA_FA_pct
Omega3
Omega6_3_ratio
Phe
PL_VSVLDL_pct
Val
CE_sLDL_pct
AcAc
Acetone
FC_sHDL_pct
Tyr
CE_sHDL
TG_CM_XLVLDL
BHB
TG_VLDL_L_pct
Lac

Figure S1. Feature selection using LASSO-Cox regression and its stability assessment. (A) Harrel's concordance measure (C-index) for mortality was assessed across various levels of penalty (lambda, λ). Optimal lambda.1se values and higher values were determined for each aging clocks: BioAge with $\lambda = 0.0166$ and 10 variables selected; Light BioAge with $\lambda = 0.0202$ and 4 variables selected; ProtAge with $\lambda = 0.018$ and 23 variables selected; MetAge with $\lambda = 0.0025$ and 27 variables selected. For ProtAge and MetAge, the right plots showed how the delta C-index varied with the number of selected biomarkers in the Lasso-Cox model. The blue dots marked the number of markers included in GOLD ProtAge and MetAge, achieving a higher C-index while balancing model complexity and performance. (B) Biomarker stability was quantified by their selection frequency across 100 replicates and bootstrapped samples. For GOLD BioAge and GOLD Light BioAge, red-highlighted biomarkers indicated features that were consistently integrated into the biological ageing clocks for mortality prediction. For GOLD ProtAge and GOLD MetAge, selected biomarkers were listed as the vertical axis to count the frequency. The abbreviations of metabolomics biomarkers were explained in Supplementary Table 9.

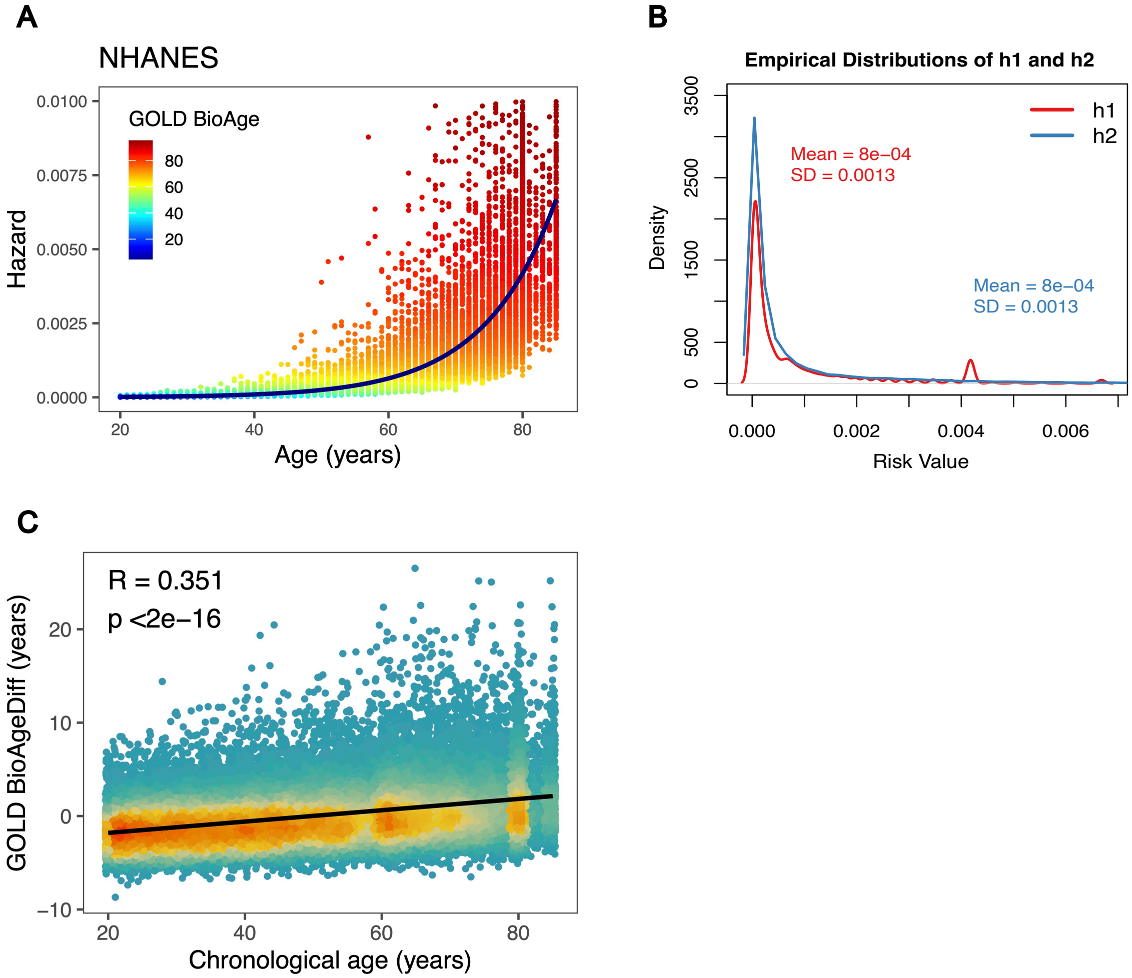

Figure S2. Estimate of GOLD BioAge and BioAgeDiff through Gompertz mortality hazard. Two Gompertz regression models (Methods, Model 1, Model 2) were used to predict mortality hazards for each individual in NHANES (A). The mortality hazards, considering both chronological age and biomarkers, were depicted as colorful points (Model 2), while the navy line represented the exponential increase of mortality hazard with chronological age (Model 1). GOLD BioAge was defined as the age corresponding to the mortality hazard of the points. The empirical density distribution of mortality hazard of the two models were shown (B). GOLD BioAgeDiff was calculated as the differences between the mortality hazard values of the line and points, showing a positive correlation with age (B).

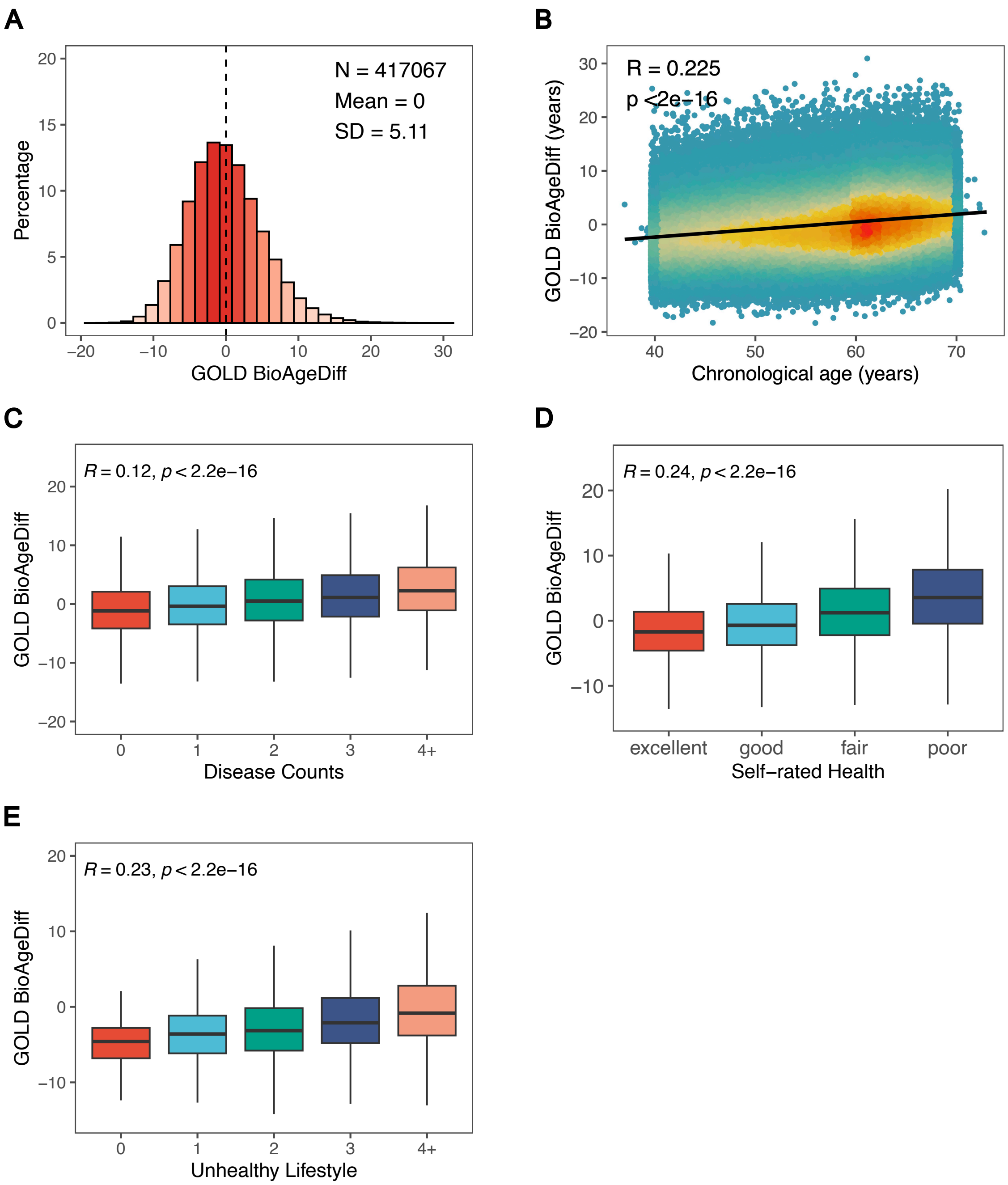

Figure S3. Validation of GOLD BioAge in UKB. The histogram (A) showed the distribution of GOLD BioAgeDiff in the UK Biobank (N = 417,067), with a mean of 0 and standard deviation of 5.11. Scatter density plot (B) showed a positive correlation of GOLD BioAgeDiff with chronological age (R = 0.225). Correlation of GOLD BioAgeDiff with health-related factors (C-E): disease counts (R = 0.12), Self-rated health (R = 0.24), and unhealthy lifestyles (R = 0.26).

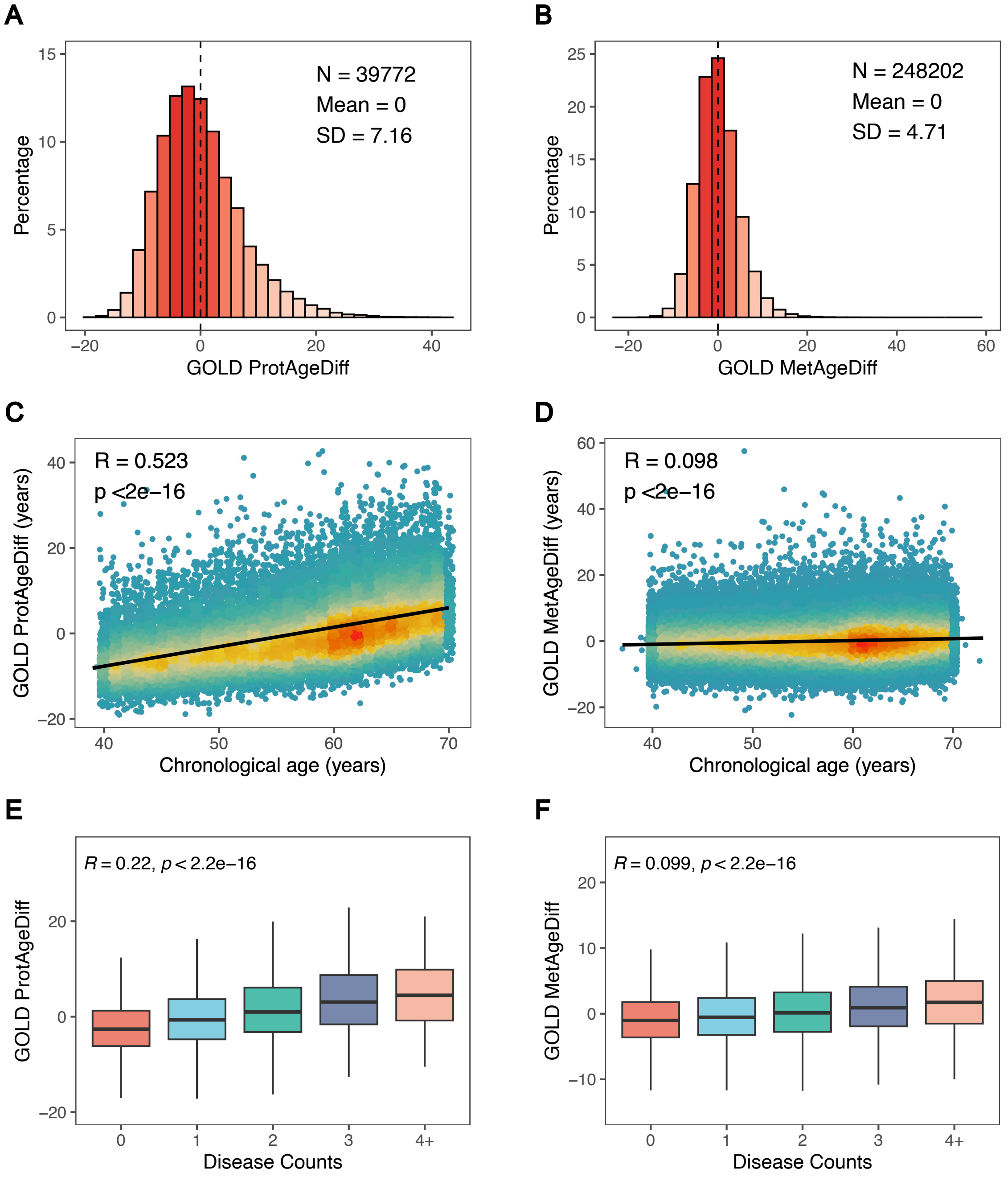

Figure S4. GOLD ProtAgeDiff and MetAgeDiff in UKB. The histograms (A-B) illustrated the distribution of GOLD ProtAgeDiff (N = 39,772) with a mean value of 0 and a standard deviation (SD) of 7.16, and GOLD MetAgeDiff (N = 248,202) with a mean value of 0 and a SD of 4.71 in the UK Biobank. Scatter density plots (C-D) showed positive correlations of GOLD ProtAgeDiff, MetAgeDiff with age (R = 0.523, 0.098, respectively). correlations of GOLD ProtAgeDiff and MetAgeDiff with chronic disease counts (R = 0.22, 0.099, respectively).

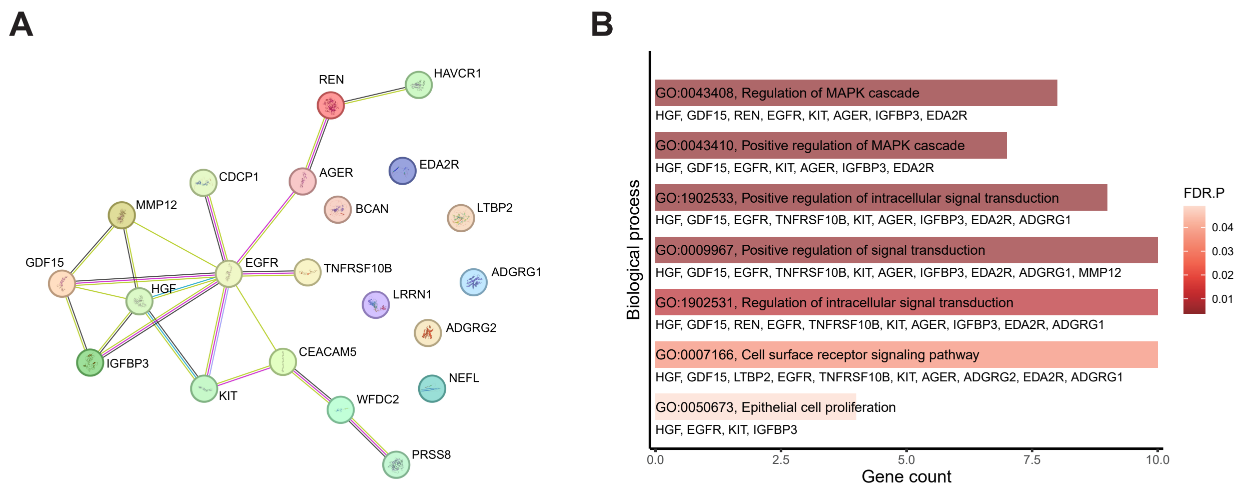

Figure S5. Protein-protein interaction (PPI) network and functional annotation of ProtAge-associated proteins. (A) PPI network edges depicted molecular interactions, with node colors denoting distinct functional clusters. (B) Significantly enriched biological processes (vertical axis) and their annotated genes (horizontal annotations).

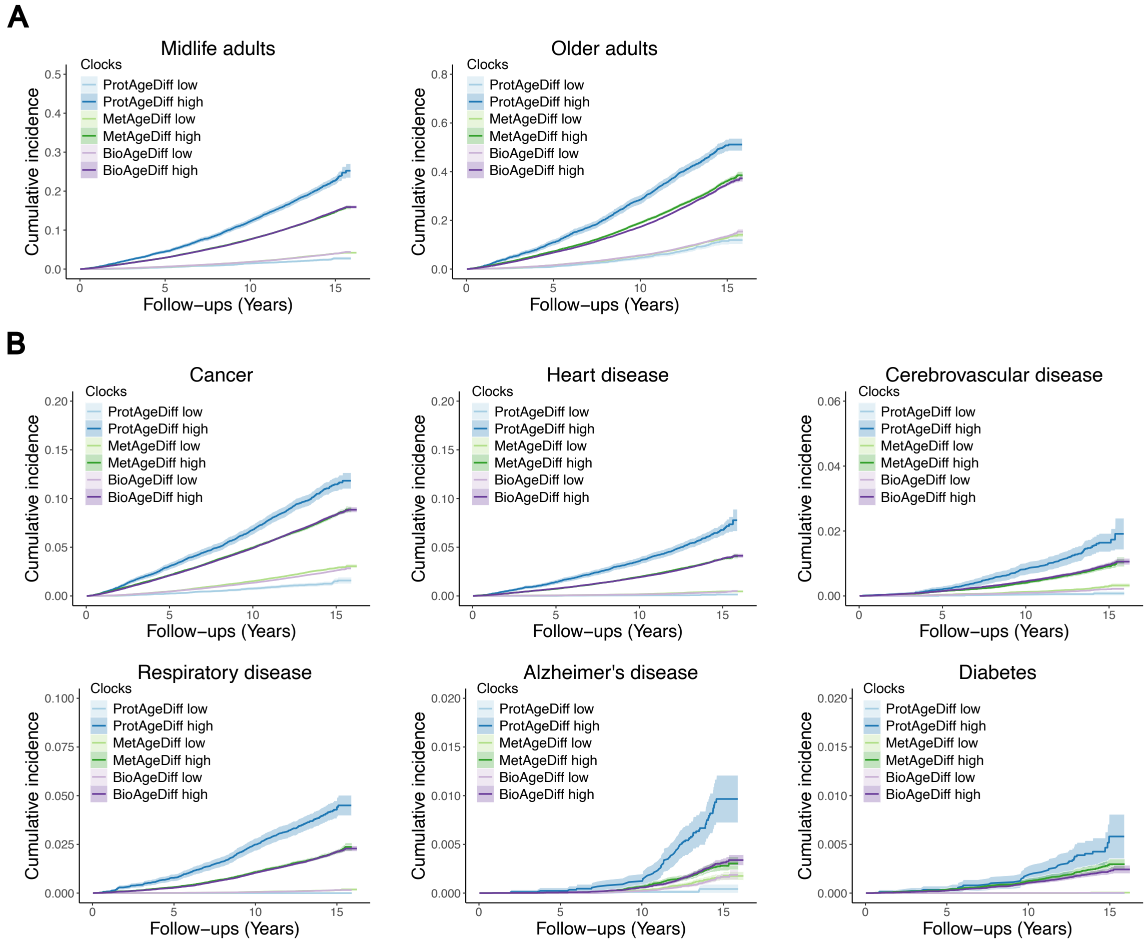

Figure S6. Associations of ProtAgeDiff, MetAgeDiff, and BioAgeDiff with mortality in UKB. The survival plots displayed the mortality trends of high-risk and low-risk groups over a follow-up period of approximately 16 years. The high-risk and low-risk categories were the top and bottom 25% of the differences of estimated biological age (ProtAge, MetAge and BioAge) and chronological age in the population. The cumulative incidences were investigated among midlife and older adults (A), and cause-specific mortality (caused by age-related chronic diseases) were also presented (B).

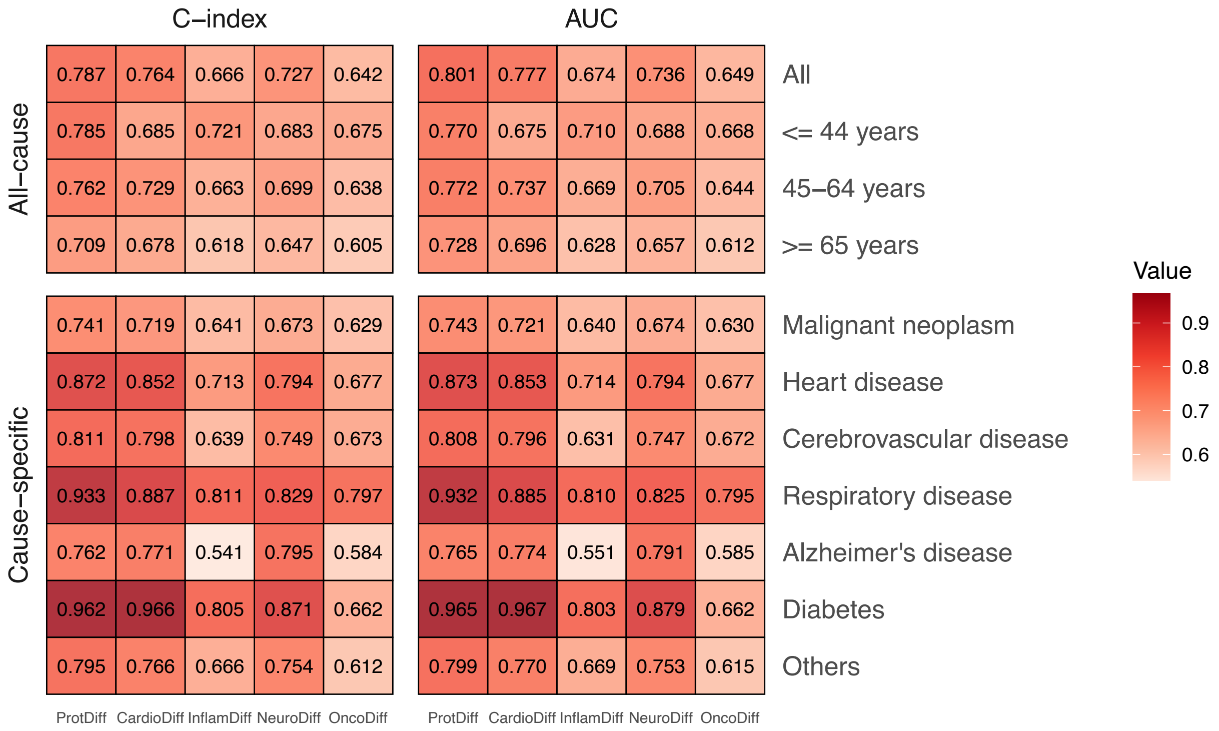

Figure S7. The predictive capability of ProtAge and its subpanels regarding mortality. The analysis included C-index in survival analysis and AUC value of 10-year mortality prediction for all-cause (age-stratified) and cause-specific mortality. The highest values were highlighted in bold. The terms ProtDiff, CardioDiff, InflamDiff, NeuroDiff, and OncDiff represented the composite scores of all proteins, as well as those specific to cardiometabolic, inflammation, neurology, and oncology categories in ProtAge.

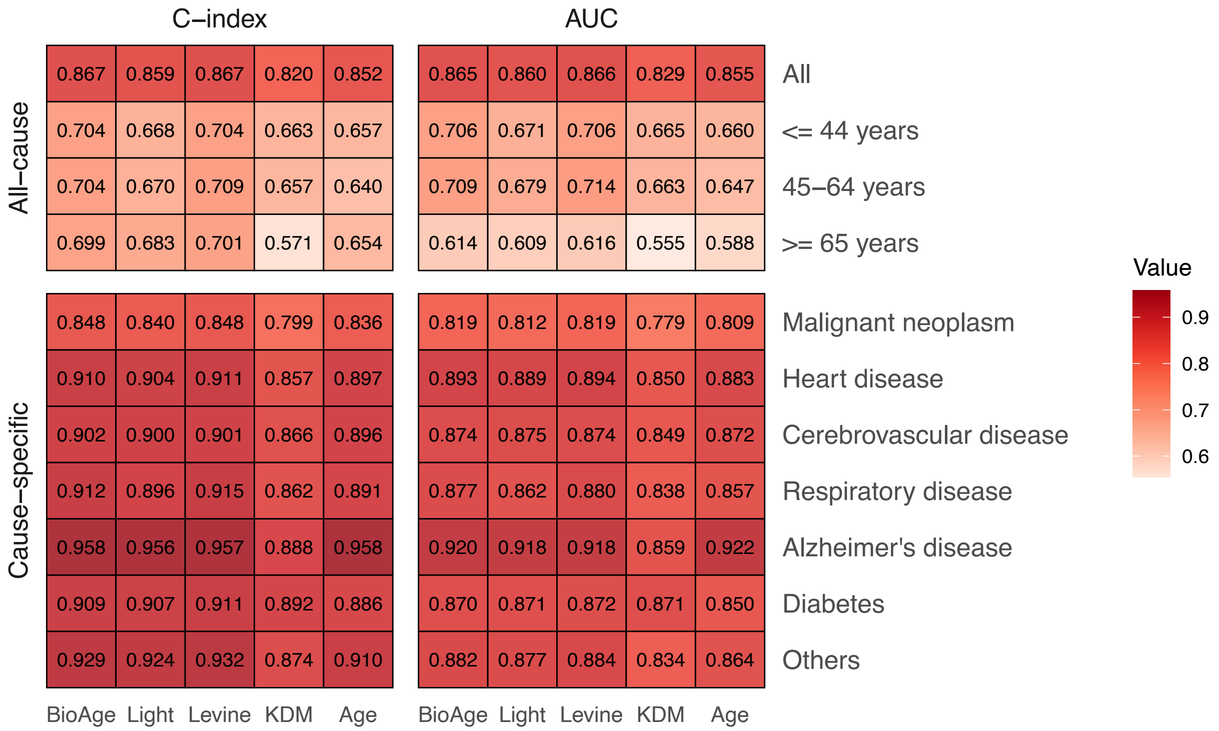

Figure S8. The comparison of GOLD BioAge, and other common aging clocks in predicting mortality in NHANES III. The evaluation included the C-index in survival analysis and the AUC value for predicting 10-year mortality using these aging clocks and chronological age for both all-cause (stratified by age) and cause-specific mortality. The highest values were denoted in bold. The BioAge, Light, Levine, and KDM referred to GOLD BioAge, its lighter version, Levine's phenotypic age, and the age derived from the KDM algorithm, respectively.

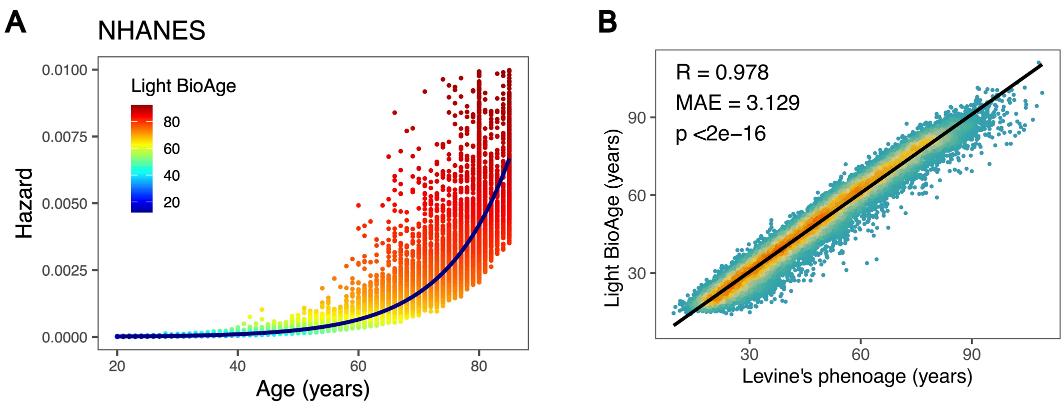

Figure S9. The mortality hazard, Light BioAge and Levine's phenotypic age in NHANES. Similar to GOLD BioAge, Light BioAge (A) indicated the age on the exponentially increasing line (Model 1) that corresponded with the mortality hazard of the colorful points (Model 2). The scatter density plot (B) revealed a strong correlation between Light BioAge and Levine's phenotypic age, demonstrating its validity as a simple measurement of biological aging.

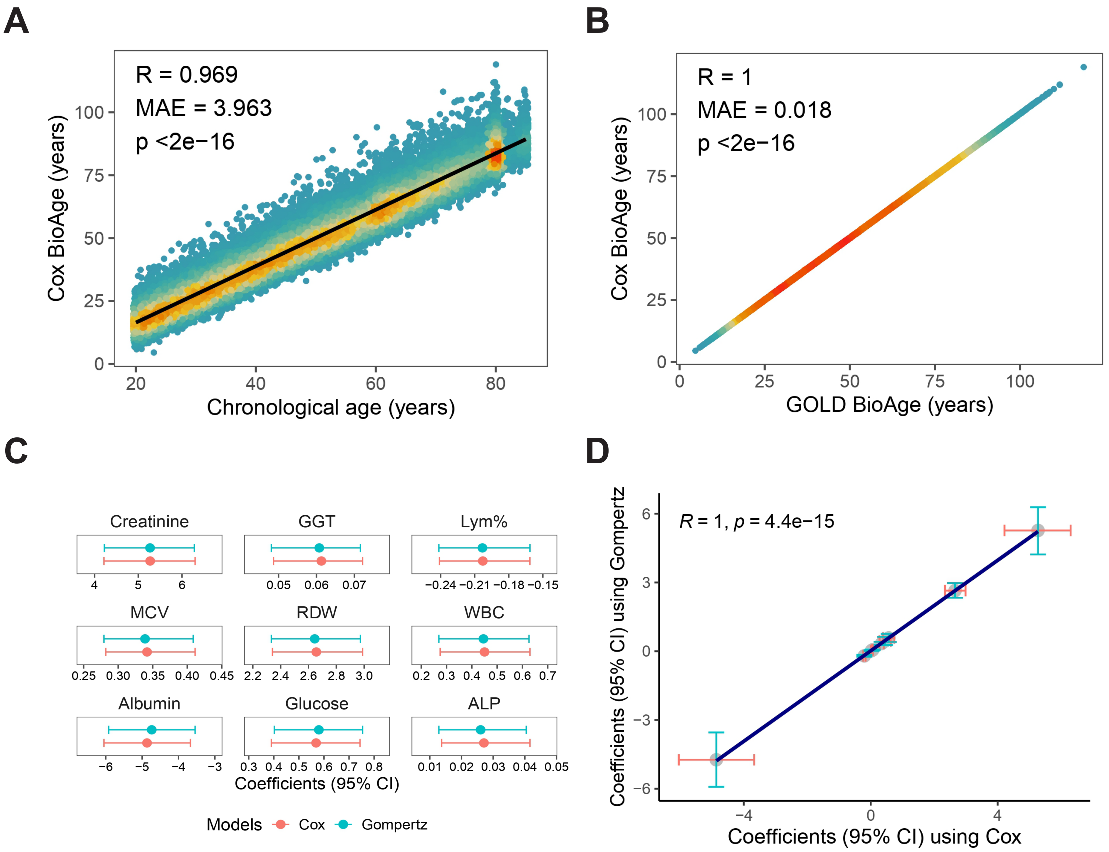

Figure S10. Comparative analysis of GOLD BioAge and Cox BioAge. (A) Correlation of Cox BioAge (derived from Cox regression) with chronological age. (B) Correlation between Cox BioAge and GOLD BioAge. (C-D) Comparison of estimated coefficients for nine biomarkers between GOLD BioAge and Cox BioAge models.
