# Supplementary material for: Gompertz Law‐Based Biological Age (GOLD BioAge): A Simple and Practical Measurement of Biological Ageing to Capture Morbidity and Mortality Risks
Source: Adv Sci (Weinh). 2025 Jul 2;12(32):e01765. doi: 10.1002/advs.202501765 (PMC12407260; doi:10.1002/advs.202501765)
Supplement: Supplementary file 3 — Supporting Information [file ADVS-12-e01765-s002.docx]

**Supplementary methods of**

Meng Hao et al. Gompertz Law-Based Biological Age (GOLD BioAge): A Simple and Practical Measurement of Biological Ageing to Capture Morbidity and Mortality Risks

**Derivation of the GOLD BioAge through Gompertz model**

The Gompertz regression, widely utilized for modeling mortality data, is primarily parameterized as a proportional hazards model[1]. The Gompertz distribution is the two-parameter function with shape parameter $a$ and rate parameter $b$. Its probability density function and hazard function are as follows:

Density function: $f\left( t|a,b \right)=be^{at}exp(-b/a(e^{at}-1))$ ,

Hazard function: $h\left( t|a,b \right)=be^{at}$ .

The hazard is increasing when shape $a>0$ and decreasing for $a<0$. According to the Hazard function, the cumulative hazard model is

$H\left( t \right)=b/a(e^{at}-1)$ .

And the cumulative distribution function of the Gompertz model is

$F\left( t|a,b \right)=1-exp(-b/a(e^{at}-1))$ .

The Gompertz model’s hazard function, when incorporating covariates such as age (CA) and biomarkers, is formally defined as:

$h\left( t|a,b \right)=bexp(\beta_{1}\cdot CA + \sum\beta_{i}*{Biomarker}_{i}+at)$.

The Gompertz model assumes that the rate of risk acceleration (parameter a) is constant across all individuals in the population. This means that: (1) Age and biomarkers exclusively influence the baseline hazard rather than risk acceleration patterns; and (2) Population-wide homogeneity exists in risk acceleration trajectories, without individual-level variation.

In GOLD BioAge framework, two parametric proportional hazard model analysis based on the Gompertz distribution were fitted. One was regressed on chronological age (Model 1); another one was regressed on selected clinical biomarkers and chronological age to estimate the mortality risks (Model 2). The hazard functions were as followed:

*Model 1:* $h_{1}\left( t, age \right)=b_{1}exp\left( a_{1}t+\beta_{1}*age \right)$ ,

*Model 2:* $h_{2}(t, age,biomarkers)=b_{2}exp(a_{2}t+\beta_{2}*age+\sum\beta_{i}*Biomarkers)$.

We defined the GOLD BioAge as the age reflecting the actual hazard risk. Therefore, we let the equation $h_{1}(t=0, GOLD BioAge)=h_{2}(t=0, age,biomarkers)$. Since the baseline hazards $h_{1}$ and $h_{2}$ differed slightly between the two models, we introduced a correction factor ($\gamma$) to adjust fror the bias, assuming $h_{1}={\gamma*h}_{2}$, where $\gamma=\frac{h_{1}}{h_{2}}$. Accordingly, the GOLD BioAge was calculated using the following formula:

$GOLD BioAge=\frac{1}{\beta_{1}}(\beta_{2}*age+\sum\beta_{i}*Biomarkers+log(\frac{\gamma*b_{2}}{b_{1}}))$ ,

To further simplifiy the above formula, we fixed the coefficient of chronological age in Model 2 equal to that in model 1 (i.e., $\beta_{2}=\beta_{1}$). Thus, the formula simplifies to:

$GOLD BioAge=age+ \frac{1}{\beta_{1}}\sum\beta_{i}*Biomarkers+\frac{1}{\beta_{1}}*log(\frac{\gamma*b_{2}}{b_{1}})$ .

The item $\frac{1}{\beta_{1}}*\ln\left( \frac{\gamma*b_{2}}{b_{1}} \right)$ was estimated by the mean value of $\frac{1}{\beta_{1}}*\log\left( \frac{{h_{1}}^{'}}{{h_{2}}^{'}}*\frac{b_{2}}{b_{1}} \right)$, where ${h_{1}}^{'}$ and ${h_{2}}^{'}$ represent the empirical values of the mortality hazard in Model 1 and Model 2, respectively. If the correction factor γ is omitted and $h_{1}\approx h_{2}$ is assumed, this constant term simplifies to $\frac{1}{\beta_{1}}*\ln\frac{b2}{b1}$. However, when incorporating γ (where $h_{1}={\gamma*h}_{2}$), this term becomes: $\frac{1}{\beta_{1}}*\log\frac{\gamma*b2}{b1}$, effectively adding $\frac{1}{\beta_{1}}*\log\gamma$ to the equation. In theory, if γ = 1, the additional term becomes 0, implying no difference between $h_{1}$ and $h_{2}$. However, empirical data from NHANES showed that γ = 1.75 ± 0.81, contributing an average of approximately 4.68 years (i.e., above 0) to the GOLD BioAge calculation. Thus, omitting γ would systematically underestimate biological age relative to chronological age in the population.

**Gompertz vs. Cox Regression**

Gompertz Regression is a proportional hazards (PH) model with a parametric baseline hazard function. A PH model’s hazard function is defined as:

$h\left( t|X \right)=h_{0}\left( t \right)\exp\left( \sum\beta_{i}*X_{i} \right)$,

where the hazard ratio between two individuals depends only on their covariates (not time). This proportional hazards assumption holds for both Gompertz and Cox models.

In the **Gompertz model**, the hazard function is:

$h\left( t|X \right)=h_{0}\left( t \right)\exp\left( \sum\beta_{i}*X_{i} \right)$,

where $h_{0}\left( t \right)=rate*\exp\left( shape*t \right)$, defines the parametric baseline hazard, exponentially increasing/ decreasing with time. The covariate effect $exp(\sum\beta_{i}*X_{i})$ scales the baseline hazard multiplicatively. For two individuals with covariates $X_{1}$ and $X_{2}$, the hazard ratio is

$\frac{h\left( t|X_{1} \right)}{h\left( t|X_{2} \right)}=exp({\sum\beta_{i}*{(X}_{1}-X}_{2}))$,

which remains constant over time, thereby satisfying the PH assumption.

Cox regression is a **semi-parametric** survival model. The hazard function for an individual at time t is given by:

$h\left( t|X \right)=h_{0}\left( t \right)exp(\sum\beta_{i}*X_{i})$ ,

where $h_{0}\left( t \right)$ is an unspecified (non-parametric) baseline hazard. Like the GOLD BioAge framework, Cox models can estimate biological age by comparing two nested models: Model 1: includes only chronological age, Model 2: includes both age and biomarkers.

*Model 1:* $h_{1}\left( t|age \right)=h_{0}\left( t \right)exp\left( \beta_{1}*age \right)$ ,

*Model 2:* $h_{2}(t|age,biomarkers)={h_{0}\left( t \right)}^{'}exp(\beta_{2}*age+\sum\beta_{i}*{Biomarker}_{i})$ *.*

Since $h_{0}\left( t \right)$ is unspecified, risks are quantified via hazard ratios (HRs):

*Model 1:* ${HR}_{1}(t|age)={h_{1}\left( t|age \right)}/{h_{0}\left( t \right)}$ ,

*Model 2:* ${HR}_{2}(t|age,biomarkers)={h_{2}(t|age,biomarkers)}/{{h_{0}\left( t \right)}^{'}}$ *.*

Let the equation ${HR}_{1}\left( t | BioAge \right)\approx{HR}_{2}\left( t | age,biomarkers \right)$. Then,

$BioAge\approx\frac{1}{\beta_{1}}\left( \beta_{2}*age+\sum\beta_{i}*{Biomarker}_{i} \right)$ .

To simplify, we set $\beta_{2}= \beta_{1}$, i.e., fix the age coefficient in Model 2 equal to that in Model 1. To adjust for bias, a variable was introduced to adjust bias, let ${HR}_{1}={\gamma*HR}_{2}$. Then,

$BioAge=age+\frac{1}{\beta_{1}}\sum\beta_{i}*Biomarkers+\frac{1}{\beta_{1}}*log\frac{{HR}_{1}}{{HR}_{2}}$ .

The $\frac{1}{\beta_{1}}*log\frac{{HR}_{1}}{{HR}_{2}}$ is took as a constant item and estimated as the mean value of $\frac{1}{\beta_{1}}*log\frac{{{HR}_{1}}^{'}}{{{HR}_{2}}^{'}}$, where ${{HR}_{1}}^{'}$ and ${{HR}_{2}}^{'}$ are the empirical values calculated by Model 1 and Model 2, respectively. The biomarker coefficients for biological age estimated using the Gompertz and Cox regression models were highly consistent, resulting in complete correlated biological ages in the NHANES. Moreover, both models demonstrated identical predictive performance for mortality, with a concordance index (C-index) of 0.869 for both the Gompertz and Cox models.

**Feature selection using Lasso-cox regression**

We used LASSO-Cox regression for feature selection, and conducted fivefold cross-validation to select the penalization parameter (𝜆). Both model performance and number of independent variables were jointly considered to develop a sparse phenotypic age estimator. For GOLD BioAge and Light BioAge, we chose lambda.1se, the value of 𝜆 that gave the most regularized model whose cross-validated error was within one standard error of the minimum. For ProtAge and MetAge, we selected values of 𝜆 slightly larger than lambda.1se to enforce stricter feature selection.

To evaluate the robustness of the final biomarker set in the GOLD BioAge framework, we performed stability analyses under two conditions: (1) sensitivity to data perturbations and (2) consistency across subsamples. For the first, we implemented fivefold cross-validation, partitioning 80% of the data into training sets and 20% into test sets. LASSO-Cox regression was applied through 100 iterations of randomized fivefold cross-validation to assess the stability of selected biomarkers under data variability. For subsample stability, we repeated LASSO-Cox regression on 100 bootstrapped subsamples, each generated by randomly sampling with replacement (maintaining the original sample size). The regularization parameters (λ) were kept consistent across all iterations to ensure comparability with baseline models. For GOLD BioAge and Light BioAge, λ optimized as lambda.1se through cross-validation across replicates and bootstraps. For GOLD ProtAge and MetAge, fixed regularization scheme with λ = exp(-4) and exp(-6) across bootstraps, respectively.

**Estimation of the GOLD BioAge**

We applied a Lasso penalized Cox regression model to select variables for constructing the Gompertz law based biological age (GOLD BioAge) using the NHANES training data. The mortality hazard was regressed on twenty-six clinical biomarkers and chronological age, with fivefold cross-validation to select the parameter value lambda (𝜆) for the penalized regression. Both the model performance and number of independent variables were considered to develop a sparse phenotypic age estimator. Finally, we chose lambda.1se, the value of 𝜆 that gave the most regularized model such that the cross-validated error was within one standard error of the minimum.

Consequently, the GOLD BioAge model involved 10 indicators, namely chronological age, creatinine, glucose, mean cell volume (MCV), red cell distribution width (RDW), albumin, alkaline phosphatase (ALP), lymphocyte percent (LYM), white blood cell count (WBC), gamma glutamyl transferase (GGT). The formula for GOLD BioAge in NHANES was as followed:

$$GOLD BioAge=Age +5.2691*Creatinine +0.5797*Glucose +0.3389*MCV$$

$$+2.6445*RDW-4.7358*Albumin+0.0260*ALP$$

$$-0.2032*LYM +0.4459*WBC +0.0608*GGT-53.6287$$

In the UKB, we updated the coefficients, considering that the two population were different. The coefficients of the formula were as followed:

$$GOLD BioAge=Age +5.3832*Creatinine +1.4168*Glucose+0.4206*MCV$$

$$+3.3162*RDW-5.0793*Albumin+0.0385*ALP$$

$$-0.1899*LYM+0.9120*WBC+0.1007*GGT-78.6519$$

Moreover, for clinical practice simplicity, we developed a much lighter version (Light BioAge), which involved chronological age, creatine, glucose, and log-transformed C-reactive protein (CRP). The Light BioAge was constructed using the following equation:

$$Light BioAge=Age+8.3313*Creatinine+0.8270*Glucose$$

$$+5.7305*Log CRP-13.5298$$

In the UKB, the formula of Light BioAge was as followed:

$$Light BioAge=Age+9.0873*Creatinine+1.7311*Glucose$$

$$+4.1689*Log CRP-20.1395$$

For external validations, the NHANES-derived Light BioAge formula was applied to the CHARLS, RuLAS, and CLHLS cohorts.

**Comparison with Levine’s Phenotypic age**

Levine[2] et al. estimated Phenotypic Age (Levine Phenotypic Age) reflecting the 10-year cumulative mortality risk (with time *t = 120* months), based on the Gompertz cumulative distribution function. Notably, $F\left( t=120,PhenoAge \right)=10-year Mortality risk$. Two Gompertz proportional hazard models were constructed as followed:

$$F_{1}\left( t=120,PhenoAge \right)=1-exp(-exp(age*\overset{^}{\beta}) *(\exp(120*a)-1/a)$$

$$F_{2}\left( t=120,X \right)=1-exp(-exp(X\overset{^}{\beta}) *(\exp(120*a)-1/a)$$

where $\overset{^}{\lambda}=X\overset{^}{\beta}$. By solved the equation $F_{1}(120, PhenoAge)=F_{2}(120, age,biomarkers)$. The Gompertz coefficients of $F_{1}$and $F_{2}$ were estimated separately.

The Phenotypic Age model is given by

$$Phenotypic Age=141.50225+\frac{ln\left( -0.00553*ln\left( 1-F_{2}(120,X) \right) \right)}{0.090165}$$

Where the mortality risk $F_{2}(120, X)$ was calculated through the Gompertz cumulative distribution function, with the linear predictor X*β* defined as:

$$X\beta=-19.907-0.0336*albumin+0.0095*creatinine +0.0195*glucose+0.0954*\log\left( CRP \right)-0.0120*LYM+0.0268*MCV+0.3356*RDW+0.00188*ALP+0.0554*WBC+0.0804*age$$

Considering that existing Gompertz-based biological age such as Levine Phenotypic Age[2-3] and Kuo’s proteomic age[4] rely on the linear combination of involved variables (X*β*), we simplified the calculation by directly utilizing the Gompertz hazard function. In both frameworks, the 10-year mortality risk (based on the cumulative distribution function) and the instantaneous mortality hazard (based on the hazard function) are derived from Xβ. However, cumulative mortality risk varies dynamically over 10 years, whereas the baseline hazard better reflects the immediate mortality risk. The calculation of GOLD BioAge showed the simplicity of our model, highlighting its efficiency to capture mortality risk through a linear combination of clinical biomarkers.

**Assessment of health- related factors and outcomes**

1. **Definition of Unhealthy Lifetyle Factors**

Unhealthy lifestyle score was based on six modifiable lifestyle factors: smoking, alcohol consumption, physical activity, diet, body mass index (BMI), and sedentary behavior. All these factors were defined consistent with World Health Organization[5-11]. They were obtained through structured questionnaires and 24-hour dietary recalls. Unhealthy BMI was defined lower than 18.5 kg/m^2^ or high than 24.9 kg/m^2^. Information on alcohol intake was self-reported. Low-risk alcohol assumption was determined as moderate drinking: no more than 2 drinks one day according to the dietary guidelines in the UK and US (one drink contains 8 g in the UK and 14 g ethanol in the US). In US NHANES, unhealthy smoke was defined as more than 100 cigarettes in a lifetime. Physical activity levels were assessed using metabolic equivalent scores. Participants were classified into thirds and the score lower than the top thirds of the population were defined as unhealthy people. Diet quality was assessed by the Alternate Mediterranean Diet Score (aMED). We defined a healthy diet as the aMED higher than the median intake. The higher score indicated a healthier diet. Sedentary behavior was defined unhealthy if the duration higher than the upper quartile of distribution (Q4). In UK Biobank, unhealthy smoke was defined as no current smoking. Poor physical activity was defined as engaging in less than 75 minutes of vigorous activity or 150 minutes of moderate activity per week (or neither of these criteria were met), or failing to engage in vigorous activity at least once per week or moderate activity at least 5 days per week[5b]. For diet, an high-risk level was defined as failing to meet the intake goals for 2 or more out of the following 3 components: (a) total fruits and vegetables ≥ 4.5 pieces or servings per day (1 serving being 3 tablespoons); (b) total fish intake ≥2 times per week; (c) red meat intake: ≤ 5 times per week and processed meat intake ≤ 2 times per week. The detrimental sedentary behavior, following previous studies, was defined as the duration of computer and television (TV) watching time more than 3 hours per day. The participants were assigned scores ranging from 0 to 4, with 0 indicating no unhealthy lifestyles and 1, 2, 3, or 4 representing individuals with one or more unhealthy lifestyle factors. We explored the association between GOLD - based biological age difference and unhealthy lifestyle score in both NHANES 2007-2010 and UKB 2006-2010. For instance, in the UKB, the distribution of unhealthy lifestyle scores was as follows: 0 (n = 3, 746), 1 (n = 31, 720), 2 (n = 75, 054), 3 (n = 96, 519), and 4 or more (n = 100, 480) (**Supplementary Table 17**).

**Comorbidity**

We assessed the number of comorbidities, which included the following disease diagnoses. The count of comorbid conditions was categorized into five groups: no disease, 1 disease, 2 diseases, 3 diseases, and 4 or more diseases. In the NHANES dataset, the conditions included diabetes, high blood pressure, congestive heart failure, coronary heart disease, heart attack, stroke, cancer or malignancy, and chronic bronchitis. In UKB, conditions considered were cancer, myocardial infarction, heart failure, stroke, chronic obstructive pulmonary disease (COPD), and dementia. For instance, in UKB, the distribution of GOLD BioAgeDiff, Light BioAgeDiff, MetAgeDiff, and ProtAgeDiff by comorbidity count was as follows: for GOLD BioAgeDiff (n = 270, 226): 0 (n = 172, 373), 1 (n = 74, 874), 2 (n = 18, 776), 3 (n = 3, 607), and 4+ (n = 596); for Light BioAgeDiff (n = 277, 431): 0 (n = 176, 975), 1 (n = 76, 871), 2 (n = 19, 276), 3 (n = 3, 698), and 4+ (n = 611); for GOLD MetAgeDiff (n = 160, 743): 0 (n = 102, 277), 1 (n = 44, 637), 2 (n = 11, 315), 3 (n = 2, 154), and 4+ (n = 360); for GOLD ProtAgeDiff (n = 25, 457): 0 (n = 16, 108), 1 (n = 7, 093), 2 (n = 1, 834), 3 (n = 362), and 4+ (n = 60).

For self-rated health, there were four levels recorded across two cohorts: excellent or very good, good, fair and poor. For example, GOLD BioAgeDiff was stratified by self-rated health levels among 417,067 participants in UKB: excellent (n = 68,432), good (n = 240,389), fair (n = 87,219), and poor (n = 18,529) (**Supplementary Table 17**).

**UK Biobank data processing**

Mortality was determined based on the recorded time of death in the UK Biobank death records (field 40000). Heart failure was identified as the first reported occurrence of heart failure (field 131354). Dementia was defined as the first reported occurrence of all - cause dementia (field 42018). Stroke was determined by the earliest reported stroke diagnosis (field 42006). Myocardial infarction (MI) was defined by the earliest reported event (field 42000). Chronic obstructive pulmonary disease (COPD) was identified based on the first reported occurrence of COPD (field 42016) ot a doctor’s report of COPD - related condition (field 22130). Cancer was identified based on the first reported occurrence of cancer (field 40005) or a doctor’s report confirming a cancer diagnosis (field 2453).

As for lifestyles, A healthy diet was defined using the following components: (a) total fruits and vegetables intake (fields 1309, 1319, 1289, 1299); (b) total fish intake (fields 1329, 1339); and (c) red and processed meat intake (fields 1349, 1369, 1379, 1389). Dietary data were collected through 24-hour online recall questionnaires sent to UK Biobank participants after their initial visit. Smoking status was obtained from fields 1239 and 1249. Physical activity levels were derived from fields 884, 894, 904, and 914. Sedentary behavior was assessed based on the TV and computer screen time, recorded in fields 1070 and 1080. Total weekly alcohol consumption was calculated as the sum of five types of alcoholic beverages: red wine (field 1568), white wine or champagne (field 1578), beer or cider (field 1588), spirits or liqueurs (field 1598), and fortified wine (field 1608). BMI was obtained from field 21001.

The biomarkers used to construct GOLD BioAge were obtained from the following UK Biobank field: creatinine (CREA, field 30700), serum glucose (GLU, field 30740), mean cell volume (MCV, field 30040), red blood cell distribution width (RDW, field 30070), albumin (ALB, field 30600), alkaline phosphatase (ALP, field 30610), lymphocyte percentage (LYM, field 30180), white blood cell count (WBC, field 30000), and gamma glutamyl transferase (GGT, field 30730). For Light BioAge, the biomarkers include creatinine (CREA, field 30700), serum glucose (GLU, field 30740), and log-transformed C-reactive protein (Log CRP, field 30710).

**References**

[1] E. T. Lee, J. Wang, *Statistical methods for survival data analysis*, John Wiley & Sons, **2003**.

[2] M. E. Levine, A. T. Lu, A. Quach, B. H. Chen, T. L. Assimes, S. Bandinelli, L. Hou, A. A. Baccarelli, J. D. Stewart, Y. Li, E. A. Whitsel, J. G. Wilson, A. P. Reiner, A. Aviv, K. Lohman, Y. Liu, L. Ferrucci, S. Horvath, *Aging (Albany NY)* **2018**, *10* (4), 573, <https://doi.org/10.18632/aging.101414>.

[3] Z. Liu, P. L. Kuo, S. Horvath, E. Crimmins, L. Ferrucci, M. Levine, *PLoS Med* **2018**, *15* (12), e1002718, <https://doi.org/10.1371/journal.pmed.1002718>.

[4] C. L. Kuo, Z. Chen, P. Liu, L. C. Pilling, J. L. Atkins, R. H. Fortinsky, G. A. Kuchel, B. S. Diniz, Aging Cell 2024, 23 (8), e14195, https://doi.org/10.1111/acel.14195.

[5] D. G. A. Committee, HHS, O. o. D. Prevention, H. Promotion, USDA, C. f. N. P. Promotion, Dietary guidelines for Americans 2015-2020, Government Printing Office, 2015;

[6] H. Xie, J. Li, X. Zhu, J. Li, J. Yin, T. Ma, Y. Luo, L. He, Y. Bai, G. Zhang, X. Cheng, C. Li, Cardiovasc Diabetol 2022, 21 (1), 199, https://doi.org/10.1186/s12933-022-01632-3;

[7] Y. Zhan, Z. Yang, Y. Liu, F. Zhan, S. Lin, BMC Public Health 2023, 23 (1), 620, https://doi.org/10.1186/s12889-023-15478-1;

[8] Y. Li, P. F. Xia, T. T. Geng, Z. Z. Tu, Y. B. Zhang, H. C. Yu, J. J. Zhang, K. Guo, K. Yang, G. Liu, Z. Shan, A. Pan, JAMA Netw Open 2023, 6 (7), e2323584, https://doi.org/10.1001/jamanetworkopen.2023.23584;

[9] X. Li, X. Cao, J. Zhang, J. Fu, M. Mohedaner, Danzengzhuoga, X. Sun, G. Yang, Z. Yang, C. L. Kuo, X. Chen, A. A. Cohen, Z. Liu, J Am Geriatr Soc 2024, 72 (1), 181, https://doi.org/10.1111/jgs.18611;

[10] Y. B. Zhang, C. Chen, X. F. Pan, J. Guo, Y. Li, O. H. Franco, G. Liu, A. Pan, BMJ 2021, 373, n604, https://doi.org/10.1136/bmj.n604;

[11] H. Han, Y. Cao, C. Feng, Y. Zheng, K. Dhana, S. Zhu, C. Shang, C. Yuan, G. Zong, Diabetes Care 2022, 45 (2), 319, https://doi.org/10.2337/dc21-1512.
